# Uncovering a new mechanism of ischemic stroke: a study of the association between γδ T cells and immunoinflammation

**DOI:** 10.3389/fimmu.2025.1583274

**Published:** 2025-07-17

**Authors:** Xuan Sun, Jiayan Wang, Hao Gu, Maojuan Guo, Zhen Yang

**Affiliations:** ^1^ Guang’an Men Hospital, China Academy of Chinese Medical Sciences, Beijing, China; ^2^ College Traditional Chinese Medicine, Tianjin University of Traditional Chinese Medicine, Tianjin, China; ^3^ Data Center of Traditional Chinese Medicine, China Academy of Chinese Medical Sciences, Beijing, China; ^4^ School of Integrative Medicine, Tianjin University of Traditional Chinese Medicine, Tianjin, China; ^5^ School of Chinese Materia Medica, Tianjin University of Traditional Chinese Medicine, Tianjin, China

**Keywords:** neurology, stroke, γδ T cells, microglia, immunity model category

## Abstract

Ischemic stroke, characterized by high clinical mortality and poor prognosis, has been prioritized by the World Health Organization (WHO) for reducing the burden of non-communicable diseases. However, the pathogenesis of ischemic stroke remains complex and poorly understood. Recent studies have revealed the infiltration of γδ T cells within ischemic stroke lesions, accompanied by the upregulation of IL-17, IL-23, and other inflammatory cytokines, suggesting their involvement in the stroke’s pathological process. Literature indicates that γδ T cells are recruited to the lesion site by microglia-derived chemokines and subsequently infiltrate the damaged brain tissue. This review summarizes current knowledge on the precise mechanisms underlying γδ T cell activation, migration, and ensuing immune-inflammatory responses in neuroinflammation, as well as their role in the progression of ischemic stroke. It further discusses the therapeutic potential of targeting γδ T cells to modulate neuroinflammation for ischemic stroke treatment, thereby offering novel therapeutic targets for managing neuroinflammation in this condition.

## Graphical Abstract

This figure illustrates a cascade of immune responses mediated by γδ T cells subsequent to cerebral ischemic stroke. Section 1: Neurons compromised by brain injury secrete signaling entities that stimulate the activation of microglia. The activated microglia subsequently undergo phenotypic polarization, differentiating into M1 (pro-inflammatory) and M2 (anti-inflammatory) phenotypes. Section 2: γδ T cells are activated by M1-polarized microglia, while concurrently, M2-polarized microglia exert an inhibitory effect on their activation. Additionally, neurons with compromised integrity release chemokines that facilitate the migration of activated γδ T cells toward the lesioned region. Section 3: Upon reaching the site of injury, γδ T cells summon neutrophils and monocytes, culminating in disruption of the blood-brain barrier and compromising the integrity of normal brain neurons and white matter.

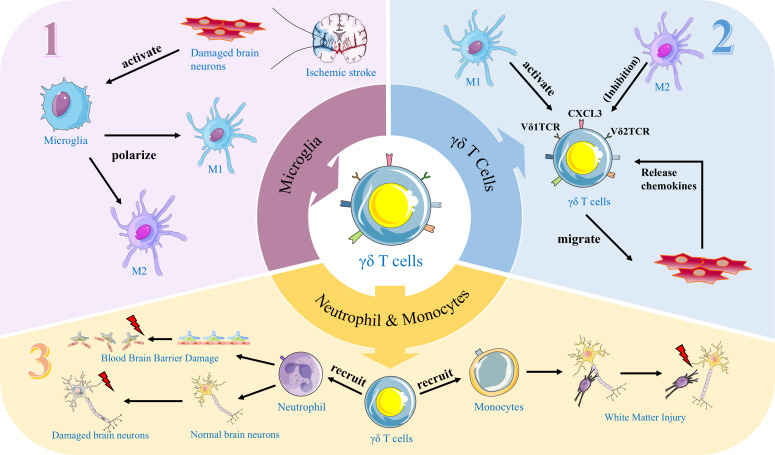

## 1 Introduction

Stroke is a serious neurological disease characterized by the sudden onset of clinical syndromes and focal or global brain dysfunction, primarily caused by vascular lesions, with symptoms lasting more than 24 hours, often resulting in disability or death (1, 2). Ischemic stroke is predominant type of stroke. The pathological feature of ischemic stroke is cerebral vascular occlusion, accounting for approximately 80%-85% of all strokes. Its global burden continues to increase, placing significant pressure on social economies and healthcare systems (**Table 1**) (3, 4). Although hypertension (5), poor diet, and aging are major risk factors (6–8), current treatment options remain significantly limited.

**Table 1:** Epidemiology and etiology of stroke.

Epidemiology and etiology	Main factors and rates
Increase in the global burden of stroke	Stroke events 70.0%
	Stroke deaths 44.0%
	Stroke prevalence 86.0%
	DALY 32.0%
16 main risk factors for stroke	High systolic blood pressure 56.8%
	Ambient particular matter 16.6%
	Cigarette smoking 13.7%
	High LDL cholesterol 13.1%
	Household air pollution 11.2%
	Diet high in sodium 10.6%
	High fasting plasma glucose 10.3%
	Kidney disfunction 9.3%
	Diet low in fruits 5.9%
	High alcohol use 5.2%
	Low temperature 4.8%
	High BMI 4.7%
	Secondhand smoking 4.4%
	Low physical activity 2.1%
	Diet low in vegetables 1.6%
	High temperature 1.1%

Due to the rapid onset of ischemic stroke, timely, accurate, and effective medical decision-making is essential to prevent long-term disability and complications (9). The current core of ischemic stroke treatment is rapid vascular recanalization, including intravenous thrombolysis within 4.5 hours of symptom onset (such as recombinant tissue plasminogen activator alteplase) and endovascular mechanical thrombectomy within 6 hours (10). Although these methods can reduce the risk of disability, they have strict time window limitations and risks of complications such as intracranial hemorrhage (11, 12), limited overall efficacy, often poor prognosis, and techniques (such as mechanical thrombectomy) are highly dependent on operator experience. Therefore, a deeper understanding of the pathological mechanisms is key to developing more effective treatments. Oxidative damage, calcium overload, and inflammatory responses induced by ischemia-reperfusion injury synergistically exacerbate brain damage (13). Therefore, an in-depth analysis of its pathological mechanisms is key to developing more effective therapies. Given that inflammatory injury persists throughout the disease course and the limited efficacy of current thrombolytic therapies combined with anti-inflammatory drugs, it is urgent to explore new targeted anti-inflammatory mechanisms to provide novel strategies for the treatment of ischemic stroke.

Experimental evidence indicates that various immune cells and lymphocytes participate in the onset and progression of ischemic stroke. Notably, the post-stroke immune response exhibits significant spatiotemporal dynamics and complexity, involving both the immune environment within the central nervous system (CNS) and peripheral immunity (14). Following stroke onset, microglia within the CNS are the first to be activated and polarized, releasing inflammatory cytokines and chemokines (15).his process simultaneously recruits peripheral immune cells to the lesion site, where they exert pro-inflammatory effects that exacerbate disease progression. γδ T cells, a distinct subset of peripheral innate lymphocytes, have garnered increasing attention. In ischemic stroke injury, they are recruited from the periphery to the CNS, leading to their activation and infiltration (16–20). Cytokines secreted by activated γδ T cells further recruit neutrophils and monocytes/macrophages to the lesion area, significantly amplifying intracerebral inflammatory damage (21–24).

However, compared to the extensive understanding of the role of γδ T cells in tumor immunotherapy, research on their function in ischemic stroke remains insufficient. Therefore, this review focuses on inflammation regulation to explore the central role of γδ T cells in the pathogenesis of ischemic stroke. Given the temporal-spatial specificity of γδ T cells in stroke-induced immunoinflammation namely, their time-dependent dynamics across different pathological stages and their spatial distribution and migration within the lesion – this article specifically examines the roles γδ T cells play during distinct phases of ischemic stroke. It further analyzes how they interact with other central and peripheral immune cells, collectively contributing to disease progression and driving inflammatory responses that exacerbate ischemic injury. This analysis aims to clarify the temporal transformation characteristics of γδ T cells across different pathological stages of ischemic stroke and their spatial migration/recruitment patterns within the immunoinflammatory context, while preserving an understanding of their involvement in processes within signaling networks during disease progression. The ultimate goal is to provide a theoretical basis for developing multi-target intervention strategies based on precise spatiotemporal modulation of γδ T cells, and to offer novel insights and approaches for the clinical treatment of ischemic stroke.

## 2 γδ T cells involved in ischemic stroke

### 2.1 Classification of mouse and human γδ T cells

Empirical evidence suggests that the ontogenetic origins of γδ T lymphocytes in murine and human species are not conserved, with each exhibiting distinct phenotypic attributes. In the murine paradigm, γδ T lymphocytes are derived from the thymic microenvironment and represent the inaugural T cell population to emerge within the embryonic thymus, with initial detection occurring as early as embryonic day 15 of murine gestation (25). In stark contrast, the presence of human γδ T lymphocytes is first ascertainable in the fetal hepatic tissue as early as 5–6 weeks into gestation (26, 27). The classification of γδ T lymphocytes is predicated upon the differential expression of T cell receptor (TCR) γ chains, including Vγ2, Vγ3, Vγ4, Vγ5, Vγ8, and Vγ9, as well as δ chains, encompassing Vδ1, Vδ2, Vδ3, and Vδ5 (28). In the murine model, γδ T cell subsets are delineated by the variability of TCR Vγ chain usage, with a predominance of Vγ4^+^ and Vγ6^+^ γδ T cells. In humans, however, γδ T cell subsets are primarily distinguished by the expression of Vδ chains, predominantly featuring Vδ1^+^ and Vδ2^+^ γδ T cells (29). The functional dichotomy of Vδ1^+^ (mucosal-resident) and Vδ2^+^ (blood-circulating) γδ T cells dictates their distinct contributions to post-stroke neuroinflammation: Vδ2^+^ cells dominate early Interleukin-17 (IL-17)-driven neutrophil recruitment, while it is assumed that Vδ1^+^ subsets may modulate late-stage repair via gut-derived metabolites (30, 31).

Most current single-cell RNA sequencing (scRNA-seq) studies have not identified γδ T cells because their transcriptomes at the single-cell level are unknown. However, there are publications that demonstrate the specific detection of human γδ T cells by high-resolution clustering of large scRNA-seq datasets and the combination of gene signatures in fresh tumor samples, allowing for the identification of their T cell receptor (TCR) Vδ1 and TCR Vδ2 subpopulations within large datasets derived from complex cellular mixtures (32–34). Furthermore, recent literature has introduced a TCR module scoring strategy for the identification of human γδ T cells, allowing for the determination of γδ T cell populations within the human body (35). This indicates that γδ T cells do indeed exist in the human body and can be subdivided at least into these two major subtypes based on their TCRs. The differentiation of human γδ T cells is influenced by tissue type and the specific γδ TCRs they express (**Table 2**). Different types of γδ T lymphocytes can be formed; for instance, Vγ9 pairs with the Vδ2 chain to create Vγ9Vδ2 T cells, which are predominantly found in peripheral blood. Conversely, γδ T cells that express the Vδ1 chain can pair with various γ chains, resulting in a range of γδ T cells in the bloodstream (36, 37).

**Table 2:** Subsets of mouse and human γδ T cells.

Subset	Paired TCR δ/γ chains	Cellular localization
Mouse γδ T cells		
Vγ1	None	Lymphoid tissue, liver
Vγ4	Vδ4	Lymphoid tissue, lung, liver, dermis
Vγ5-DETC	Vδ1	Epidermis
Vγ6	Vδ1	Uterus, lung, tongue, liver
Vγ7	None	Intestinal mucosa
Human γδ T cells		
Vδ1	Vγ2, Vγ3, Vγ4, Vγ5, Vγ8 and Vγ9	Skin, intestine, liver, spleen and mucosal tissues
Vδ2	Vγ9	Peripheral blood
Vδ3	Vγ2, Vγ3	Liver and peripheral blood
Vδ5	Vγ4	Peripheral blood

**Figure 1:**
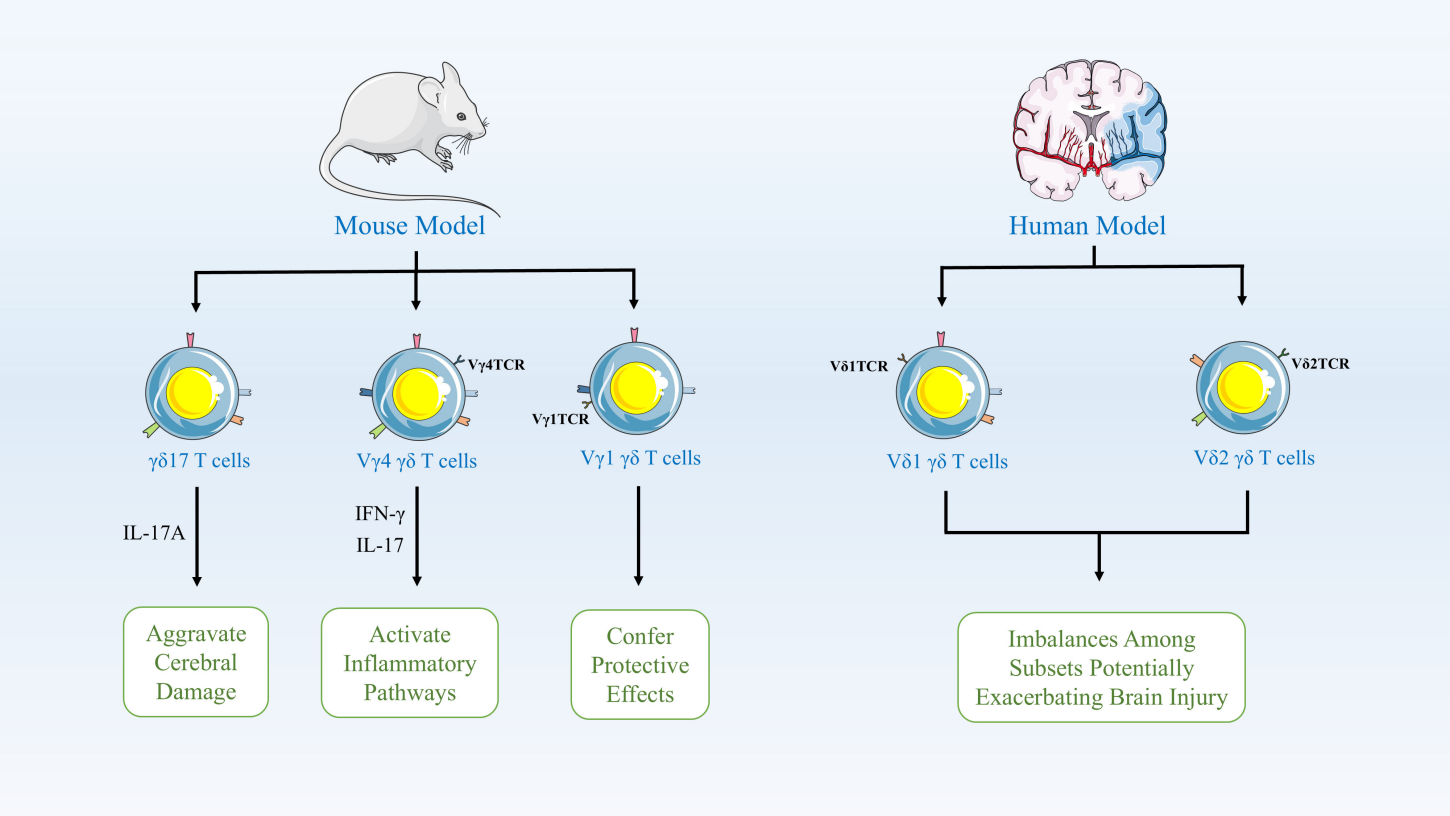
γδ T Cell Subsets in Ischemic Stroke Pathophysiology. In mouse models, γδ T cells critically modulate stroke outcomes. The γδ17 subset rapidly infiltrates the brain early post-stroke, releasing IL-17A to amplify neuroinflammation and injury. Pro-inflammatory Vγ4 cells secrete IFN-γ/IL-17, activating damaging brain inflammatory pathways, while Vγ1 cells may be protective. In humans, γδ T cell dynamics correlate with disease severity and recovery. Reduced Vδ2 subset frequency during acute stroke associates with worse neurological status (higher impairment scores) and poor long-term functional outcomes. γδ T cells regulate post-stroke immune dysregulation across phases; subset imbalances exacerbate injury via inflammatory cascades and bone metabolism factors.

γδ T cells typically act as early responders to inflammatory lesions and are a crucial source of IL-17 and IFN-γ (38). Research indicates that γδ T17 cells are recruited to sites of inflammation 7–10 days prior to the antigen presentation required for CD4^+^ T cell activation, allowing them to initiate antigen-dependent responses earlier (38, 39). In murine models, γδ T cells play a pivotal role in the pathophysiology of ischemic stroke, with distinct subsets performing different functions. Specifically, the γδ17 T cell subset rapidly infiltrates the brain during the early phase of stroke and releases IL-17A, thereby amplifying detrimental immune responses and exacerbating brain injury (40). The Vγ4 subset secretes pro-inflammatory cytokines such as IFN-γ and IL-17, activating inflammatory pathways in the brain; these subsets primarily exert their effects by exacerbating neuroinflammation and promoting brain damage. In contrast, the less abundant Vγ1 subset may confer protection by secreting TGF-β, thereby maintaining microglial homeostasis, suppressing hyperactivated neuroinflammatory responses, and mitigating brain injury (41). Consequently, in models of ischemic injury, the major γδ T cell subsets exhibit pro-inflammatory functions, and inhibiting γδ T cells or their markers significantly reduces brain damage by lowering levels of inflammatory mediators and neuronal apoptosis, thereby improving functional outcomes (42). Furthermore, in clinical studies of ischemic stroke, alterations in γδ T cell subsets are closely associated with disease progression and recovery in patients. Research indicates that during acute ischemic stroke, a reduction in the Vδ2 subset correlates with worse neurological status, manifested as higher deficit scores and adverse clinical outcomes (43). γδ T cells participate in both acute and chronic inflammatory processes post-stroke, and a decrease in the Vδ2 subset is associated with unfavorable long-term functional recovery (43, 44). Additionally, the role of γδ T cells in stroke pathophysiology includes regulating immune dysregulation; an imbalance between subsets may indirectly exacerbate brain injury by influencing inflammatory pathways and bone metabolism-related factors (45). These data indicate that γδ T cell subsets play a key immunomodulatory role in human stroke, directly impacting neuroprotection and functional recovery (43). (**Figure 1**).

Most chemokines expressed in brain neurons during ischemic stroke can recruit γδ T cells (**Table 3**). Chemokines are categorized into four subfamilies according to their structural variations: CC, CXC, CX3C, and XC (46). Once secreted, chemokines induce directed chemotactic migration by coupling to seven-helix chemokine receptors via G proteins on the cell surface, signaling cell migration (47, 48).

**Table 3:** Chemokine and chemokine receptors related to murine γδ T.

CC/CXC	Chemokine	Chemokine receptor
CC chemokine/receptor family	CCL17	CCR4
	CCL2 (MCP-1)	CCR5
	CCL3 (MIP-1α)	CCR5
	CCL4 (MIP-1β)	CCR5
	CCL5 (RANTES)	CCR5
	CCL20	CCR6
	CCL19 (MIP-3β)	CCR7
	CCL21 (SLC)	CCR7
	CCL25 (TECK)	CCR9
	CCL27	CCR10
CXC chemokine/receptor family	CXCL5	CXCR1
	CXCL6	CXCR1
	CXCL8 (IL-8)	CXCR1
	CXCL9	CXCR3
	CXCL10 (IP-10)	CXCR3
	CXCL11	CXCR3
	CCL21 (SLC)	CXCR3
	CXCL12 (SDF-1)	CXCR4
	CXCL16	CXCR6

It was found that mRNA and protein expression of chemokine ligand 2 (CCL2) and chemokine receptor 2 (CCR2) significantly increased in the rat hippocampus 6 hours after cerebral ischemia-reperfusion injury (49). In particular, CCR5 is differentially upregulated in mRNA and protein expression in immune cells, astrocytes, and neurons during cerebral ischemia/reperfusion injury, playing a crucial role in disease progression (50). Similarly, the expression of chemokine (C-X-C motif) ligand12 (CXCL12) on the neuronal surface is upregulated after cerebral ischemic injury, while the expression of CXC chemokine receptors 4 (CXCR4) is upregulated in microglia and astrocytes, enhancing the inflammatory response to injury (51). Simultaneously inducing γδ T-cell infiltration. Therefore, these studies suggest that γδ T cells infiltrate the injury site in ischemic stroke by expressing these chemokine receptors.

γδ T cells are predominantly distributed in the intestinal lamina propria (LP) and epithelium. Specifically, γδ T cells and intestinal flora provide different signals for regulating host immune system effects or modulating phenotype (52, 53). As shown in **Table 2**, different subpopulations of human γδ T cells have been categorized (54). γδ T cells are expressed in the dermis as Vγ5 (dendritic epidermal cells) and Vγ4 TCR (skin γδ T cells) in skin inflammation, and when they migrate to the peripheral blood, they can express CCR6 and CCR2 (55–59). Additionally, Vγ1 and Vγ4 T cells develop postnatally and circulate in the lymphatic system and bloodstream (60). Studies indicate that γδ T cells originating from various sites like the intestine can migrate to the brain and could contribute to the γδ T cell population during ischemic stroke (61, 62).

### 2.2 Recruitment of γδ T cells

It has been demonstrated that γδ T cells infiltrate the brain parenchyma post-ischemic injury via chemokine gradients (e.g., CXCL12/CXCR4 axis) (63, 64). During the acute phase of ischemic stroke, levels of these chemokines are significantly elevated. Serum CXCL12 levels are elevated in patients with acute ischemic stroke, showing a positive correlation with stroke severity (65); CXCL10 is increased in brain tissue or inflammatory responses, documented as an indicator of inflammation within 48 hours post-stroke, and is associated with neurological injury (66, 67). The critical role of γδ T cells in ischemic stroke-induced brain injury primarily involves cytokines released by γδ T lymphocytes, including IL-17, IL-21, IL-22, and IFN-γ, along with cytokine-recruited immune cells (68). Understanding how γδ T cells are activated and migrate, as well as how they induce an immune-inflammatory response, is crucial in ischemic stroke research.

#### 2.2.1 Activation of M1 and M2 microglia

**Figure 2:**
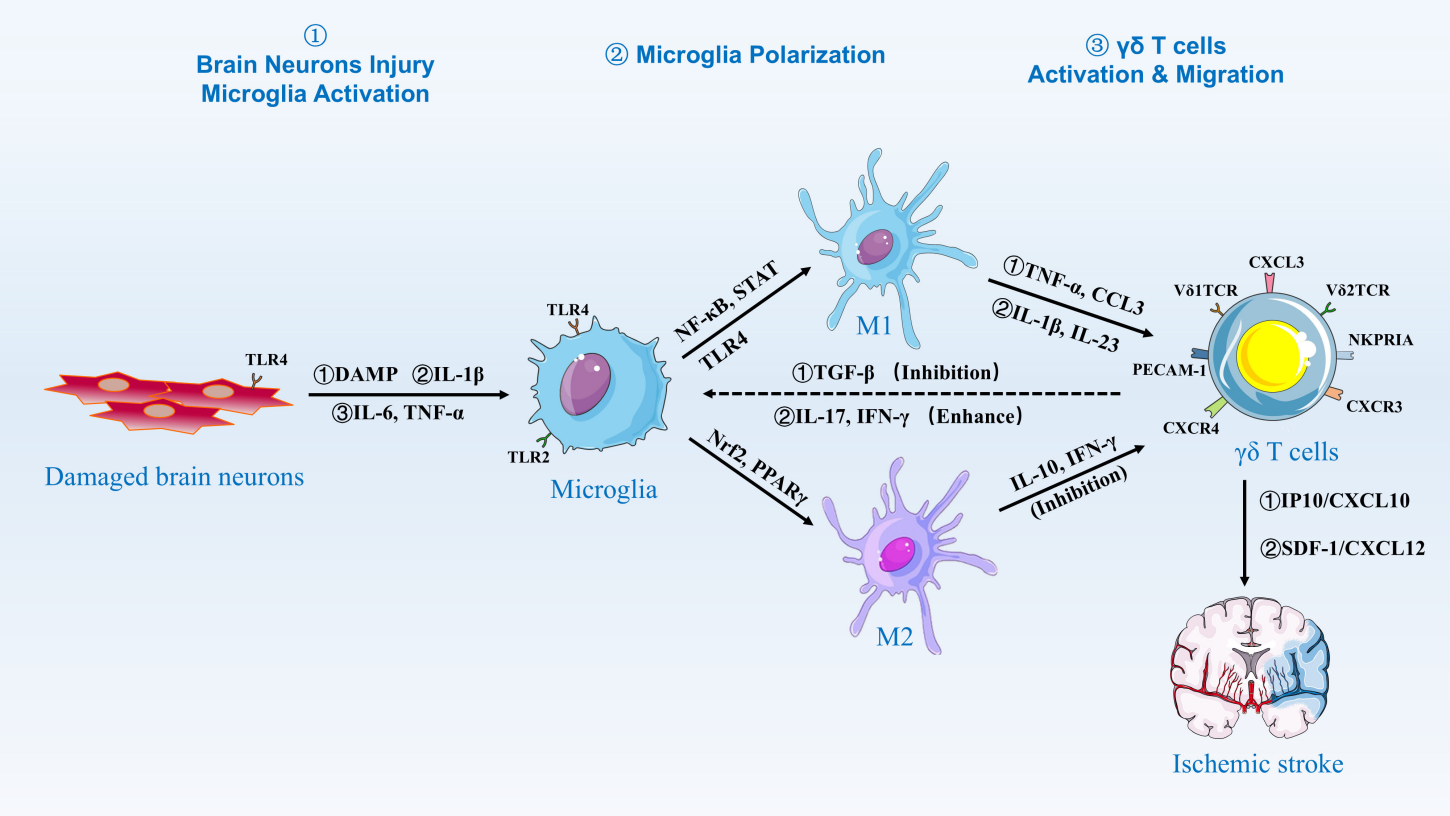
Activation and Migration of γδ T Cells by Microglia. Damaged brain neurons release DAMPs, IL-1β, IL-6, and TNF-α, which activate microglia via TLR2 and TLR4 signaling. Activated microglia then polarize into M1 and M2 phenotypes. M1 microglia are induced by NF-κB and STAT signaling, releasing pro-inflammatory cytokines such as TNF-α and CCL3, while M2 microglia are induced by Nrf2 and PPARγ signaling, releasing anti-inflammatory cytokines such as IL-10 and IFN-γ. The balance between M1 and M2 polarization is regulated by factors such as TGF-β, which inhibits M1 polarization, and IL-17 and IFN-γ, which enhance M1 polarization. γδ T cells are activated and migrate to the site of injury in response to chemokines such as IP10/CXCL10 and SDF-1/CXCL12, contributing to the inflammatory response and tissue repair in ischemic stroke.

M1/M2 microglial polarization is dynamically regulated by post-stroke inflammatory cues, Microglia are innate immune cells in the brain, constituting 5-20% of neuroglia (69, 70). As the resident macrophages within the central nervous system (CNS), microglia continuously perform immunosurveillance under normal conditions, removing microorganisms, dead cells, redundant synapses, protein aggregates, and other harmful substances, while secreting soluble factors that contribute to the immune response and tissue repair (71–73). They support normal neuronal physiological activity by providing nutritional support, removing apoptotic debris, and eliminating faulty synapses (74–77). Microglia are the first immune cells to sense ischemia and respond immediately following an ischemic stroke (21, 78, 79). Once activated and initiating the defense process, microglia enhance phagocytosis and express increased levels of receptors, cytokines, chemokines, and other inflammatory molecules, aiding in the recruitment of additional immune cells to the damaged area (80). (**Figure 2**).

Studies have shown that disruptions in brain homeostasis, such as inflammation and oxidative stress, lead to microglia activation. Following the onset of ischemic stroke, microglia are activated through damage-associated molecular patterns (DAMPs), including heat shock proteins released from necrotic cells, and non-protein alert proteins like adenosine triphosphate (ATP) (81–83). Toll-like receptors (TLRs) are key components of the innate immune system, acting as pattern recognition receptors (PRRs) that recognize pathogen-associated molecular patterns (PAMPs) and DAMPs (84). This triggers immune responses, including the release of inflammatory cytokines and activation of downstream signaling pathways. These responses play a critical role in defending against infections, regulating tissue homeostasis, and bridging innate and adaptive immunity (85, 86). In ischemic stroke, TLR2 and TLR4 are particularly crucial in regulating microglia activation and play a key role in inducing neurodegeneration (87, 88). Studies using an apoptosis-associated speck-like protein (ASC) knockout mouse model with a C-terminal caspase-activation and recruitment domain (CARD) have shown that microglia sense PAMPs and ATP released from damaged neurons (89). When microglia sense PAMPs and ATP released from injured neurons, TLRs on microglia are stimulated, leading to the formation of intracellular IRAKM-caspase-8-ASC inflammasomes that secrete ASC-dependent IL-1β. This nonclassical inflammasome-derived IL-1β can expand microglia populations through autocrine signaling (89). Conversely, when injured neurons express high levels of TLR4, it activates the NF-κB and NMDAR/PSD95-nNOS pathways, releasing proinflammatory factors such as tumor necrosis factor-alpha (TNF-α) and interleukin-6 (IL-6), which activate microglia (90, 91). Experiments in mouse models have shown that TLR2 is similarly expressed in microglia in the lesion area, and that high expression of TLR2 exacerbates ischemic stroke lesions, increasing infarct size and further amplifying stroke-induced CNS damage (92).

Similar to the aforementioned studies, the current research on the mechanisms of stroke microglia is primarily conducted using animal models, including mice and rats. Upon activation, microglia can polarize into two states: M1 and M2. These polarization states are influenced by ischemic stroke factors, including transcription factors, receptors, and ion channels (93). Among these, NF-κB, STAT family members, TLR4, S1PR3 binding to S1P, and ROS can activate M1 microglia. Activated M1 microglia then release significant amounts of cytokines and chemokines, such as TNF-α, IL-1β, IFN-γ, IL-6, inducible nitric oxide synthase (iNOS), and matrix metalloproteinases (MMP9, MMP3) (83, 94, 95). This release exacerbates inflammation and impairs the blood-brain barrier, allowing monocytes and macrophages to migrate to the damaged area, which further aggravates the inflammatory response (96). Meanwhile, M1 microglia produce free radicals and oxidants, such as those generated by NADPH oxidase, which cause oxidative stress and have deleterious effects (97). Nrf2 transcription factors and PPARγ are associated with M2 microglia activation. Upon activation, M2 microglia work in conjunction with macrophages to secrete anti-inflammatory factors, including IL-10 and transforming growth factor β (TGF-β). These factors help suppress inflammatory responses and facilitate revascularization (98–100). Additionally, M2 microglia produce trophic factors, such as insulin-like growth factor 1 (IGF-1), which promotes neuronal proliferation, differentiation, and maturation, contributing to central nervous system repair after ischemic stroke (101–105). Additionally, interactions between microglia and other immune cells, such as T cells, modify the microenvironment created by DAMPs and neural antigens, influencing the state of the inflammatory response (99). Microglial cells have been shown to significantly impact the inflammatory response to stroke.

As discussed, the M1 polarization state of activated microglia mediates the inflammatory response exacerbated by neuronal injury. Several studies have shown that the ischemic milieu is a critical factor influencing microglia function and their activation phenotype (106, 107). Therefore, it is crucial to regulate T-cell infiltration, inhibit M1 microglia activation, and promote M2 microglia polarization to mitigate inflammation, improve the metabolic state and environment of the ischemic site, and provide neuronal protection. This approach is essential for maintaining CNS homeostasis (108). Ischemic stroke is a dynamically evolving disease process, necessitating different therapeutic approaches at various stages of the disease. Intervening in the dynamic transition between M2 and M1 microglia could be a key focus for future stroke treatments. Further research is needed.

#### 2.2.2 Microglia-mediated activation of γδ T cells

Under normal conditions, microglia express a variety of scavenger receptors and TLRs as they continually monitor their environment for signs of injury or infection. As a significant component of the inflammatory response, γδ T cells, constituting 20% of total T cells, accumulate in the focal area within 24 hours after ischemic stroke, influencing the process (64, 109). Interactions between microglia and γδ T cells mainly involve the activation of γδ T cells by M1 microglial cells and the release of cytokines that either promote or inhibit microglial cell activation. The mechanism may involve TLR activation. Katja et al. demonstrated that M1 microglia activated by TLR-specific ligands upregulated CD69 and CD25, and secreted IL-17 (110). The supernatants, which contained ligands for TLR2, TLR4, TLR7, or TLR9, facilitated the activation of γδ T cells through the secretion of cytokines IL-1β and Interleukin-23 (IL-23). Microglia can induce IL-17 secretion from γδ T cells. However, M2 microglia produce IL-10, which limits IL-17A signaling (23). Within 24 hours post-ischemia, DAMPs (e.g., HMGB1) activate microglial TLR4, inducing MyD88-dependent NF-κB translocation and subsequent IL-1β/IL-23 secretion (111). These cytokines prime Vγ6^+^Vδ1^+^ γδ T cells to produce IL-17A, which peaks at 72 hours and correlates with neutrophil influx (112). By contrast, beyond day 7, TGF-β from M2 microglia suppresses γδ T cell activity, favoring resolution phases (111, 112). Additionally, it has been shown that activated γδ T cells secrete IFN-γ, which activates the microglia-A1 astrocyte-C3-neuron C3aR neurotoxicity pathway, exacerbating neuronal injury (113). Thus, microglia-γδ T cell interaction in mice stroke involves activated microglia mediating γδ T cell activation, IL-17 secretion, and mutual influence on activation states (**Figure 2**).

Inhibiting the crosstalk between microglia and γδ T cells may be crucial for reducing secondary injury induced by ischemic stroke. Administering rapamycin within 6 hours post-focal ischemia, which targets the mammalian target of rapamycin (mTOR), or employing interferon beta (IFN-β) treatment in a transient middle cerebral artery occlusion/reperfusion (tMCAO/R) mouse model, or inhibiting perforin-mediated neurotoxicity, significantly reduces the proinflammatory activity of microglia at the site of brain injury in rats. These interventions also inhibit chemokine production by microglia, thereby reducing γδ T-cell infiltration (114–116). Additionally, experiments in rat models have demonstrated that poly (ADP-ribose) polymerase (PARP) inhibitors, minocycline, or histone deacetylase inhibitors (HDACIs) such as valproic acid and sodium butyrate has been shown to effectively inhibit microglia activation when administered for sustained periods following focal ischemia. This inhibition is crucial as it correlates with an enhancement in neuronal survival, suggesting a potential therapeutic strategy for neuroprotection (117–119). These findings confirm the close relationship between microglia and γδ T cells.

## 3 γδ T cell migration

γδ T cells develop from thymocyte precursors independently of TCR signaling and are influenced by the cytokine SRY-Box Transcription Factor 13 (Sox13) (120). Studies have demonstrated that subpopulations of γδ T cells producing IFN-γ, IL-4, and IL-17 are programmed in the mouse thymus before migrating to peripheral tissues. Upon leaving the thymus, they are transported through the bloodstream to secondary lymphoid organs and then to tissues, or they return from tissues to the circulation (121, 122). γδ T cells preferentially circulate through non-lymphoid tissues by rolling on the vascular endothelium to induce specific glycoproteins, followed by selectins and integrins that promote adherence to the endothelium, resulting in leukocyte arrest (123). The lymphocytes then migrate to endothelial cells at the intercellular junctions (124).

**Figure 3:**
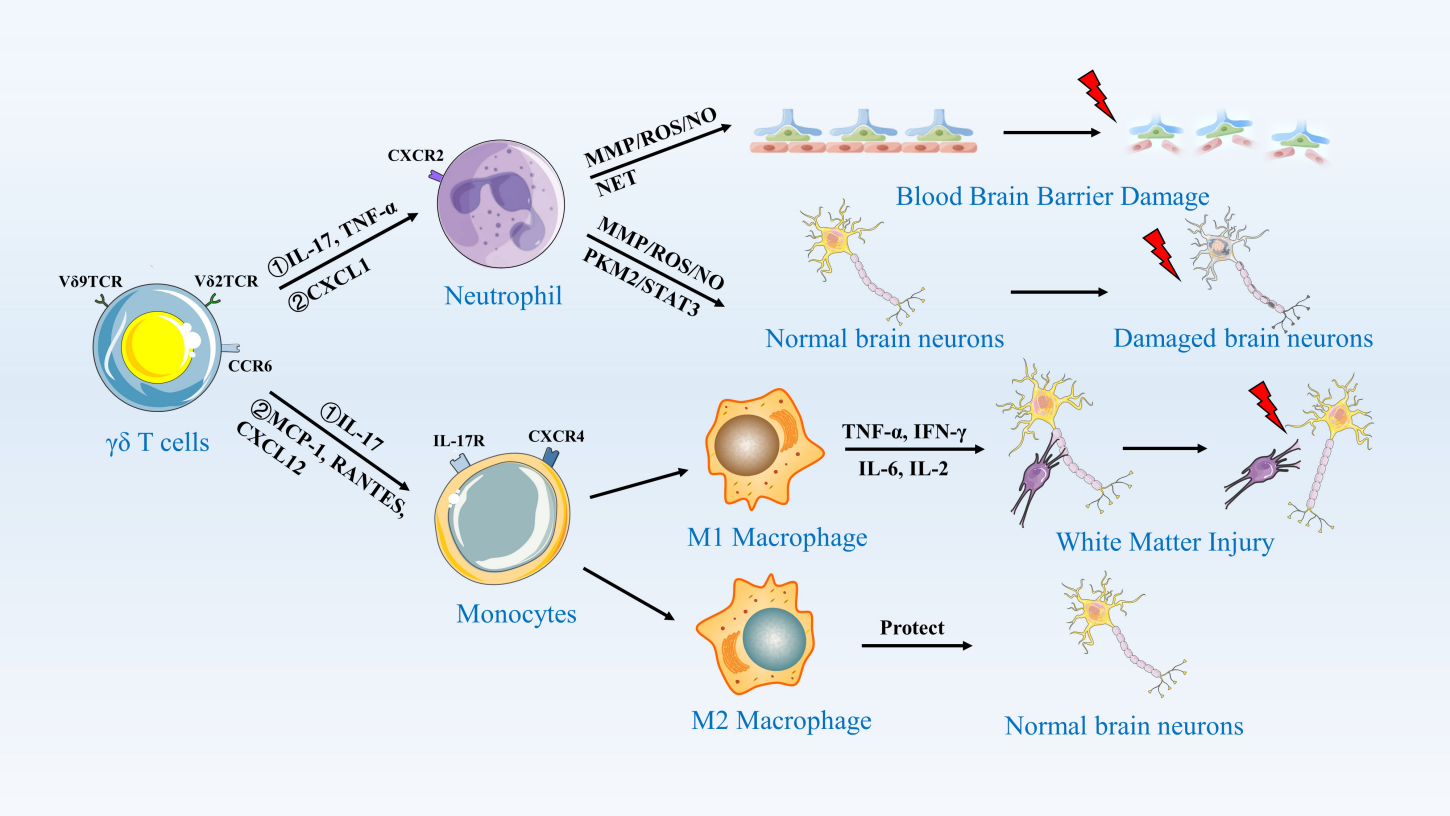
γδ T Cells Activate Neutrophils and Monocytes/Macrophages to Induce an Inflammatory Response. This figure illustrates the role of γδ T cells in central nervous system inflammation. γδ T cells, expressing Vδ9TCR and Vδ2TCR, recognize antigens and secrete cytokines such as IL-17 and TNF-α through CCR5. These cytokines activate neutrophils, which release MMP/ROS/NO and NET, leading to blood-brain barrier damage. Additionally, γδ T cells secrete MCP-1, RANTES, and CXCL12, which activate monocytes. Monocytes differentiate into M1 and M2 macrophages. M1 macrophages, through the secretion of TNF-α, IFN-γ, IL-6, and IL-2, further damage normal brain neurons, resulting in white matter injury. In contrast, M2 macrophages secrete protective factors that help maintain the integrity of normal brain neurons.

Unlike in mice, different subsets of human γδ T cells exhibit distinct patterns of migration. γδ T cells can be classified into Vδ1 and Vδ2 T lymphocytes based on the function of their δ-chain in human. Vδ1^+^ T cells are predominantly located in mucosal regions, whereas Vδ2 T cells primarily circulate in peripheral blood and lymph nodes (125, 126). Most γδ T cell subsets found at the site of ischemic stroke injury are Vγ9 and Vδ2 T cells. Therefore, it is hypothesized that in human ischemic stroke injury, γδ T cells recruited and migrating to the injury site are more likely to originate from peripheral blood and lymph nodes. Both subpopulations may undergo inflammatory changes or respond to chemokines produced by γδ T cells, with Vδ1 T cells expressing PECAM-1^+^CXCR4^+^ in response to interferon-induced protein-10 (IP10/CXCL10) and using this molecule for migration. In contrast, Vδ2 T cells express NKRPIA and CXCR3 in response to stromal-derived factor (SDF-1/CXCL12) and use it for migration in endothelial cells (127, 128). Post-ischemia, the CXCL12 gradient peaks at 24–48 hours, coinciding with γδ T cell infiltration. Intriguingly, hypoxia-inducible factor-1α (HIF-1α) stabilizes CXCL12 transcription in peri-infarct astrocytes, while endothelial CXCR4 upregulation facilitates γδ T cell arrest via β2-integrin clustering (129, 130). Pharmacological blockade of CXCR4 in murine models reduces γδ T cell transmigration by 60%, highlighting this axis as a therapeutic checkpoint (131). (**Figure 3**).

Studies suggest that circulating γδ T lymphocytes may be sensitive to chemotactic or mechanotactic cues *in vivo*, allowing them to target damaged tissues. There are also experiments in brain diseases other than stroke to prove the mechanism of cell migration and promoting inflammatory response. Infiltration of γδ T cells at the damage site has also been observed in mice with experimental allergic encephalitis (EAE) However, administration of anti-γδ TCR did not deplete TCR signaling but rather inhibited it. Conversely, early γδ T cells secrete IL-17A, which enhances late Th17 cytotoxicity, suggesting their involvement in multiple sclerosis (MS) or EAE (132). γδ T cells exhibit a multifaceted role in MS progression in human samples (133). In mice with EAE, γδ T cells infiltrate the damaged brain parenchyma through integrin β2 (134, 135). Consequently, we conclude that the migration of γδ T cells is crucial for initiating inflammation.

## 4 γδ T cells orchestrate neutrophil and macrophage-driven inflammation

### 4.1 γδ T cells activate neutrophils to induce an inflammatory response

Within 24 hours after the onset of ischemic stroke, specific γδ T cell subsets (Vγ6^+^CCR6^+^ and Vγ9^+^Vδ2^+^), upon binding to IL-17R, release IL-17A and become the primary source of IL-17A (24, 136). Their activity peaks within 3 days post-stroke and serves as a key accelerator of disease progression (137). Furthermore, IL-17A synergizes with TNF-α to activate the ACT1-TRAF6 complex in astrocytes, driving sustained NF-κB-dependent CXCL1 production (138, 139). This CXCL1 recruits CD16^+^CD62L^+^ N1 neutrophils, which release MMP-9 and ROS, exacerbating blood-brain barrier (BBB) leakage (140). This cascade results in neutrophil infiltration into the injury site, where they invade the compromised brain parenchyma and impair its function (24, 136). Depletion of γδ T cells shifts neutrophil polarization towards an N2 phenotype (CD206^+^Arg1^+^), indicating the existence of a bidirectional crosstalk exploitable for immunomodulation (141). Additionally, interferon regulatory factor 4 (IRF-4)-expressing dendritic cells are recognized as the source of IL-23, which drives and sustains IL-17 production by γδ T cells, thereby inducing the neutrophil recruitment mechanism. Consequently, depleting dendritic cells or genetically disrupting the IL-23 signaling pathway reduces IL-17 production in γδ T cells, leading to a reduction in infarct size in murine models of ischemic stroke (136, 142).

The accumulation of neutrophils recruited to the site of central nervous system injury increases the production of cytotoxic molecules, such as pro-inflammatory cytokines, matrix metalloproteinases (MMPs), reactive oxygen species (ROS), and the multifunctional protein pyruvate kinase M2 (PKM2). These molecules initially disrupt the integrity of the blood-brain barrier (BBB) and further promote neuronal lysis and apoptosis, thereby exacerbating brain injury (143, 144). Studies have documented that in murine models of ischemic stroke, inflammatory cytokines TNF-α and IL-1β, along with hypoxia-inducible factor 1-alpha (HIF-1α) activation, induce the expression of MMP-9 and MMP-2. These MMPs are recognized as the principal proteases responsible for BBB disruption, subsequently degrading the basement membrane to facilitate neutrophil infiltration into the brain parenchyma (145–147). Furthermore, stroke induction triggers the nuclear translocation of PKM2 in neutrophils, mediating thrombo-inflammatory responses via STAT3 phosphorylation, which aggravates ischemia-reperfusion injury (148). Similarly, elevated levels of ROS generated by neutrophils directly damage junctional proteins and the endothelial cytoskeleton, further exacerbating the inflammatory injury response in ischemic stroke (149).

The release of neutrophil extracellular traps (NETs) by activated neutrophils also exacerbates damage in ischemic stroke. The formation of intravascular and parenchymal NETs peaks within 3–5 days after stroke onset. Depletion of γδ T cells promotes NET formation by neutrophils, which impairs vascular remodeling and disrupts the blood-brain barrier (BBB) during recovery from ischemic stroke (150–152). In the early phase of ischemic stroke, an elevated peripheral neutrophil count is associated with larger infarct volumes and poorer clinical outcomes and prognoses (153). The neutrophil-to-lymphocyte ratio (NLR) is considered the optimal predictor of post-ischemic stroke events (153). A higher NLR upon admission in patients with acute ischemic stroke, particularly within 48 hours of symptom onset, indicates a poorer prognosis at 3 months (154). Thus, γδ T cells clearly represent a crucial mechanism for neutrophil activation that drives the inflammatory response in cerebral ischemic stroke. This reveals significant bidirectional crosstalk between γδ T cells and neutrophils, laying the groundwork for future immunomodulatory therapies targeting this pathway.

### 4.2 γδ T cells activate monocytes/macrophages to induce an inflammatory responses

During the acute phase of ischemic stroke, the likelihood of Ly6C^hi^ monocyte-derived macrophages being present in the brain is low, but the number of monocytes in the blood increases dramatically (155). After ischemic stroke, immature proinflammatory Ly6C^hi^CD43^low^CCR2 monocytes in the peripheral circulation are recruited to the brain after neutrophils and infiltrate the ischemic brain tissue to reach the core of the lesion as tissue macrophages (156). Experimentally, it has been confirmed that monocyte recruitment and macrophage infiltration are regulated through the CXCL12/CXCR4 axis (157). In ischemic muscle tissues of mice, γδ T-cell depletion has been shown to lead to an increase in the number of proinflammatory M1 macrophages (151). IL-17R is highly expressed on Ly6C^hi^ monocytes, and IL-17A is able to induce cytokines and chemokines that are trophic for monocytes, including chemotactic protein-1 (MCP-1), RANTES, and CXCL12/CXCR4, enabling splenic and circulating monocytes to migrate through the endothelium to the damaged brain parenchyma and differentiate into tissue macrophages (**Table 4**) (158–160). It was found that IL-17 levels were reduced and circulating monocyte infiltration decreased by depletion of γδ T cells (161). Specifically, γδ T cells producing IL-17A serve as a major early source of this cytokine in the acute inflammation, and their ability to rapidly respond to damage signals surpasses that of Th17 cells (162, 163). In ischemic stroke, IL-17 produced by γδ T cells and by Th17 cells exhibits significant differences in timing, function, and context. Temporally, during the acute phase of stroke, γδ T cells rapidly release IL-17A following stroke onset to amplify early detrimental immune responses (164), while Th17 cells function throughout the stroke process, including in pathogenesis, induction of secondary injury, and regulation of late-stage repair (165, 166). Functionally, IL-17A derived from γδ T cells primarily exacerbates neuroinflammation and brain injury in the acute phase by promoting neutrophil recruitment and early immune amplification, worsening ischemic damage (164, 167), whereas IL-17A produced by Th17 cells has more diverse roles, not only promoting neuroinflammation and secondary injury (165, 168), but also potentially participating in repair processes during the recovery phase (167). Contextually, IL-17A levels in γδ T cells may be directly modulated by the gut microbiota and dietary factors, reflecting their responsiveness in local microenvironments (164), while IL-17 production by Th17 cells relies on more complex regulatory mechanisms, including extracellular signals (e.g., IL-23 activation), transcription factors (e.g., RORγt), RNA, and epigenetic modifications, all of which influence their differentiation and function in the stroke microenvironment (165, 169, 170).

**Table 4:** Summary of chemokines/signaling pathways for γδ T cell-mediated monocyte recruitment.

Stage	Chemokines/pathways	Function
Peripheral Monocyte Recruitment	MCP-1/RANTESCXCL12/CXCR4	Recruits monocytes from the spleen and circulation to migrate to brain injury sites, where they differentiate into macrophages
Macrophage Recruitment	MCP-1/PR3/ICAM-1/CCL2	Enhances macrophage recruitment
Macrophage Polarization	mTORC1-S6K1 TGF-β-PPARγ	Promotes M1 polarization Promotes M2 polarization
Macrophage Inflammatory Role	JAK2/STAT3&NLRP3 CX3CR1 (High Expression)	Releases pro-inflammatory cytokines and promotes cerebral edema Macrophages undergo phenotypic switching from M1 to M2

Moreover, γδ T cell-derived IL-17A binds on monocytes, activating the mTORC1-S6K1 axis to promote M1 polarization (171). Conversely, by day 7, TGF-β from M2 microglia suppresses mTOR signaling, enabling PPARγ-driven M2 transition (172). Targeting this temporal switch with rapamycin may balance pro-inflammatory and reparative responses. Additionally, MCP-1 released by neutrophils and endothelial cells mobilizes circulating monocytes to infiltrate the site of ischemic stroke injury, and protease PR3 released by neutrophils upregulates the expression of endothelial ICAM-1 and CCL2 to enhance macrophage recruitment (173, 174). In ischemic stroke, once vascular occlusion occurs, leading to intravascular hypoxia and inducing DAMP and ROS production, the endothelium becomes less responsive to the stress response. This, in turn, stimulates the expression of cell adhesion molecules in endothelial cells, disrupting the BBB and facilitating monocyte entry into the site of injury. A vicious cycle is formed, exacerbating disease progression (175).

It has been found that macrophages transform into different phenotypes at different times during ischemic stroke and thus play different roles. Their proinflammatory effects occur mainly 2–4 days after ischemic stroke, and MCAO examination detects circulating monocytes and monocyte-derived macrophages at the site of damaged brain tissue. Macrophage polarization at the site of damage induces an M1 proinflammatory phenotype that exacerbates oligodendrocyte death and demyelination, thereby worsening cerebral white matter injury (176, 177). Recent studies demonstrate that the JAK1/2 inhibitor, Ruxolitinib, reduces the release of proinflammatory factors by inhibiting the activation of the nucleotide-binding oligomerization domain-like receptor protein 3 (NLRP3) inflammasome in macrophages, as well as the JAK2/STAT3 pathway, thereby ameliorating brain edema after stroke (**Table 4**) (178). Macrophages subsequently undergo a phenotypic switch on day 7, transforming into M2 macrophages with tissue repair and remodeling functions, losing expression of Ly6C and CCR2 but highly expressing CX3CR1 (**Table 5**) (179).

**Table 5:** Key Experimental models and findings in ischemic stroke immunopathology.

Model category	Key findings	References
Human studies		
γδ T Cells	1. Increased infiltration of γδ T cells (Vδ2^+^ subset) in ischemic brain tissue correlates with disease progression. 2. Human γδ T cell migration is mediated by the CXCL10/CXCL12-CXCR3/CXCR4 axis.	(27, 28, 30, 38)
Microglia	1. Microglia sense DAMPs (e.g., ATP) via TLR4, polarizing to a proinflammatory M1 phenotype (CD86^+^/iNOS^+^). 2. Post-stroke oxidative stress and inflammation drive microglial activation, exacerbating neuronal injury through IL-1β and TNF-α release.	(59, 60, 72)
Neutrophils	1. Elevated neutrophil-to-lymphocyte ratio (NLR) within 48 hours predicts poor 3-month outcomes. 2. Neutrophil-derived proteases (MMP9/MMP2) disrupt the blood-brain barrier (BBB), mediated by TNF-α and IL-1β.	(124, 127–129)
Murine models		
γδ T Cells	1. Vγ6^+^ γδ T cells rapidly infiltrate ischemic brain tissue within 24 hours, secreting IL-17A to recruit neutrophils. 2. IL-23 signaling sustains γδ T cell-derived IL-17 production; disrupting this pathway reduces infarct size.	(115, 135, 145)
Microglia	1. TLR2/TLR4 activation drives M1 polarization, releasing proinflammatory cytokines (TNF-α, IL-6) that disrupt the BBB. 2. Rapamycin (mTOR inhibitor) suppresses microglial inflammation and γδ T cell recruitment.	(74, 82, 101, 102)
Neutrophils & NETs	1. Neutrophil extracellular traps (NETs) peak 3–5 days post-stroke, exacerbating BBB disruption and impairing vascular repair. 2. Neutrophil depletion mitigates BBB damage and enhances post-stroke angiogenesis.	(132–134)
Rat models		
Microglia	1. CCL2/CCR2 expression surges in the hippocampus 6 hours post-ischemia, promoting monocyte infiltration. 2. M2 microglia secrete anti-inflammatory cytokines (IL-10, TGF-β) and neurotrophic factors (IGF-1) to support CNS repair.	(64, 88–91)
Therapeutic Targets	1. PARP inhibitors, HDAC inhibitors (e.g., valproic acid), and minocycline suppress microglial activation, improving neuronal survival. 2. JAK1/2 inhibition (ruxolitinib) reduces brain edema by blocking NLRP3 inflammasome activation in macrophages.	(104, 106, 160, 161)

## 5 Discussion

Neuroinflammation is a critical mechanism in ischemic stroke, involving the orchestrated participation of various immune cells that drive disease progression. This intricate immune regulation likely stems from the time-dependent (e.g., acute vs. chronic phases) and spatially specific (e.g., brain-infiltrating vs. peripherally recruited immune cells) nature of the post-stroke immune response. γδ T cells, endowed with unique innate immune properties, have emerged as pivotal initiators of neuroinflammation. During the early stages of stroke, γδ T cells primarily exert pro-inflammatory functions, while adaptive immune cells subsequently mount protective responses to curb inflammation and support neural regeneration (180–182). As rapid innate responders, γδ T cells recognize damage-associated molecular patterns (DAMPs) via TLRs, promoting microglial polarization toward the M1 phenotype. They are activated by cytokines such as IL-1β and IL-23 secreted by microglia (82, 83, 183). Chemotactically guided by CXCL10 and CXCL12, γδ T cells migrate into the ischemic region and secrete IL-17 to amplify inflammation (184). The IL-17 and CXCL12 produced by γδ T cells further drive neutrophil infiltration and monocyte/macrophage migration to the lesion site, respectively, exacerbating secondary injury and contributing to ischemic stroke progression (156, 157).

Furthermore, during ischemic stroke, γδ T cells dynamically modulate the stroke immune microenvironment through interactions with other immune cells. This includes bidirectional regulatory circuits with αβ T cells, regulatory T cells (Tregs), dendritic cells (DCs), as well as microglia and NK cells. Literature demonstrates that γδ T cells serve as a critical nexus linking the innate and adaptive immune systems during ischemic stroke (137, 185). Specifically, γδ T cells typically exacerbate acute brain injury through IL-17A production, triggering a highly conserved innate immune response in the acute phase of stroke (23, 24, 137, 142). They further synergize with αβ T cells to promote cerebral tissue damage (142, 186, 187). Concurrently, interactions between γδ T cells and Tregs influence adaptive immunity (166, 188, 189). Conversely, Tregs suppress IL-17A production by γδ T cells indirectly via IL-10 signaling, while also restricting the pro-inflammatory functions of αβ T cells through modulation of IL-10 receptor signaling (23, 189). Additionally, synergistic interactions between γδ T cells and microglia amplify neuroinflammation. For instance, co-secretion of pro-inflammatory cytokines with M1-polarized microglia contributes to secondary injury (24, 168, 190), whereas Tregs and M2-polarized microglia foster anti-inflammatory responses (168, 180, 190, 191). In summary, through interactions with other cellular subsets within the immune network, γδ T cells orchestrate complex immunomodulatory mechanisms in the ischemic stroke microenvironment (192).

Therefore, targeting γδ T cells to modulate neuroinflammation represents a novel therapeutic strategy for ischemic stroke. Studies demonstrate that blocking γδ T cells, IL-17a, or IL-21 confers significant neuroprotective effects against ischemic brain injury in murine stroke models, establishing them as promising therapeutic targets for mitigating ischemic brain damage (109, 193). Specifically, while IL-17A inhibitors (e.g., Secukinumab) are clinically used for autoimmune diseases, their application in stroke remains confined to animal studies. Conversely, γδ T cell agonists, such as α-GalCer, 5-(2-oxopropylideneamino)-6-D-ribitylaminouracil (5-OP-RU), and aminobisphosphonates, can activate immune responses under immunosuppressive conditions (194–196). Moreover, a potential link has been identified between γδ T cells and transient receptor potential (TRP) channels. TRPV1 modulates T cell activation and differentiation, which may indirectly affect γδ T cell activity (197, 198) and consequently influence post-stroke neuronal injury (199, 200). Blocking TRPV3 or TRPM2 shows potential for reducing brain damage and improving stroke outcomes (201, 202), an effect potentially linked to modulation of γδ T cell activity. This provides new perspectives on immunomodulation by regulating γδ T cell responses for ischemic stroke treatment.

Looking forward, the time-dependent and spatially specific role of γδ T cells in ischemic stroke, combined with advances in technology, holds promise for brain-targeted drug delivery using specialized encapsulation materials. This approach aims to enhance therapeutic precision and reduce peripheral side effects. Beyond pharmacological interventions, non-pharmacological approaches like dietary modifications (203–205) and electroacupuncture (206) also show efficacy in modulating immune responses. Consequently, by deepening our understanding of the inflammatory microenvironment regulation in ischemic stroke and its underlying mechanisms, we anticipate the discovery of effective novel therapeutic targets.

## 6 Conclusion

γδ T cells mediate post-stroke immunoinflammation through the TLR4/IL-17 axis, with their synergy with αβ T cells and interspecies heterogeneity presenting both therapeutic opportunities and challenges. Advancing multi-omics technologies and interdisciplinary collaboration will be critical to bridging the gap between mechanistic insights and clinical translation, ultimately enabling precision immune modulation in ischemic stroke.

To address current gaps, future research should prioritize: Cross-species mechanistic validation: Establishing humanized stroke models to compare functional heterogeneity among γδ T cell subsets. Metabolomic-epigenetic crosstalk: Combining metabolomics and chromatin accessibility profiling to elucidate how microbiota-derived metabolites (e.g., short-chain fatty acids) regulate γδ T cell plasticity via HDAC or mTOR pathways. Temporally targeted therapies: Developing phase-dependent strategies, such as acute-phase inhibition of IL-17/IL-23 signaling and recovery-phase enhancement of Treg activity to promote neural repair. Notably, multi-omics combined research overcomes the limitations of single-omics techniques, enabling a more systematic and comprehensive analysis of the complex biological behaviors and molecular mechanisms of γδ T cells in ischemic stroke, but research on γδ T cells in ischemic stroke is still in the exploratory stage. Advancing multi-omics technologies and interdisciplinary collaboration will be critical to bridging the gap between mechanistic insights and clinical translation, ultimately enabling precision immune modulation in ischemic stroke. However, such studies also face numerous challenges, such as the integration of multi-omics technologies and the complexity of data analysis, requiring the establishment of standardized procedures and methods; as well as how to better translate animal experimental results into clinical applications. Nevertheless, with continuous technological advancements and deeper research, γδ T cells are expected to become a new target for immunotherapy in ischemic stroke, bringing new hope for improving patient prognosis and quality of life.

## References

[1] Benjamin EJ Muntner P Alonso A Bittencourt MS Callaway CW Carson AP. Heart disease and stroke statistics-2019 update: A report from the american heart association. Circulation. (2019) 139:e56–e528. doi: 10.1161/CIR.0000000000000659, PMID: 30700139

[2] Sacco RL Kasner SE Broderick JP Caplan LR Connors JJ Culebras A. An updated definition of stroke for the 21st century: a statement for healthcare professionals from the American Heart Association/American Stroke Association. Stroke. (2013) 44:2064–89. doi: 10.1161/STR.0b013e318296aeca, PMID: 23652265 PMC11078537

[3] Feigin VL Brainin M Norrving B Martins S Pandian JD Lindsay P. World stroke organization (WSO): global stroke fact sheet 2025. Int J stroke: Off J Int Stroke Soc. (2024) 20(2):132–44. doi: 10.1177/17474930241308142, PMID: 39635884 PMC11786524

[4] Feigin VL Owolabi MO. Pragmatic solutions to reduce the global burden of stroke: a World Stroke Organization-Lancet Neurology Commission. Lancet Neurol. (2023) 22:1160–206. doi: 10.1016/S1474-4422(23)00277-6, PMID: 37827183 PMC10715732

[5] Feigin VL Brainin M Norrving B Martins SO Pandian J Lindsay P. World stroke organization: global stroke fact sheet 2025. Int J stroke: Off J Int Stroke Society. (2025) 20:132–44. doi: 10.1177/17474930241308142, PMID: 39635884 PMC11786524

[6] Stoll G Nieswandt B. Thrombo-inflammation in acute ischaemic stroke - implications for treatment. Nat Rev Neurology. (2019) 15:473–81. doi: 10.1038/s41582-019-0221-1, PMID: 31263257

[7] Walter K. What is acute ischemic stroke? Jama. (2022) 327:885. doi: 10.1001/jama.2022.1420, PMID: 35230392

[8] Boursin P Paternotte S Dercy B Sabben C Maïer B. Sémantique, épidémiologie et sémiologie des accidents vasculaires cérébraux [Semantics, epidemiology and semiology of stroke]. Soins. (2018) 63(828):24–7. doi: 10.1016/j.soin.2018.06.008, PMID: 30213310

[9] Mendelson SJ Prabhakaran S. Diagnosis and management of transient ischemic attack and acute ischemic stroke: A review. Jama. (2021) 325:1088–98. doi: 10.1001/jama.2020.26867, PMID: 33724327

[10] Widimsky P Snyder K Sulzenko J Hopkins LN Stetkarova I. Acute ischaemic stroke: recent advances in reperfusion treatment. Eur Heart J. (2023) 44:1205–15. doi: 10.1093/eurheartj/ehac684, PMID: 36477996 PMC10079392

[11] Lakhan SE Kirchgessner A Hofer M. Inflammatory mechanisms in ischemic stroke: therapeutic approaches. J Trans Med. (2009) 7:97. doi: 10.1186/1479-5876-7-97, PMID: 19919699 PMC2780998

[12] Fisher M Schaebitz W. An overview of acute stroke therapy: past, present, and future. Arch Internal Med. (2000) 160:3196–206. doi: 10.1001/archinte.160.21.3196, PMID: 11088079

[13] He Y Wang J Ying C Xu KL Luo J Wang B. The interplay between ferroptosis and inflammation: therapeutic implications for cerebral ischemia-reperfusion. Front Immunol. (2024) 15:1482386. doi: 10.3389/fimmu.2024.1482386, PMID: 39582857 PMC11583640

[14] Barthels D Das H. Current advances in ischemic stroke research and therapies. Biochim Biophys Acta Mol basis disease. (2020) 1866:165260. doi: 10.1016/j.bbadis.2018.09.012, PMID: 31699365 PMC6981280

[15] Du O Wu C Yang YX Yang HY Wu YJ Li MY. High mobility group box 1, a novel serotonin receptor-7 negative modulator, contributes to M2 microglial ferroptosis and neuroinflammation in post-stroke depression. Free Radic Biol Med. (2025) 237:666–83. doi: 10.1016/j.freeradbiomed.2025.06.025, PMID: 40527447

[16] Kipnis J. Multifaceted interactions between adaptive immunity and the central nervous system. Sci (New York NY). (2016) 353:766–71. doi: 10.1126/science.aag2638, PMID: 27540163 PMC5590839

[17] Iadecola C Anrather J. The immunology of stroke: from mechanisms to translation. Nat Med. (2011) 17:796–808. doi: 10.1038/nm.2399, PMID: 21738161 PMC3137275

[18] Hammond TR Marsh SE Stevens B. Immune signaling in neurodegeneration. Immunity. (2019) 50:955–74. doi: 10.1016/j.immuni.2019.03.016, PMID: 30995509 PMC6822103

[19] Vantourout P Hayday A. Six-of-the-best: unique contributions of γδ T cells to immunology. Nat Rev Immunol. (2013) 13:88–100. doi: 10.1038/nri3384, PMID: 23348415 PMC3951794

[20] Li Y Zhang Y Zeng X. γδ T cells participating in nervous systems: A story of jekyll and hyde. Front Immunol. (2021) 12:656097. doi: 10.3389/fimmu.2021.656097, PMID: 33868300 PMC8044362

[21] Wang S Zhang H Xu Y. Crosstalk between microglia and T cells contributes to brain damage and recovery after ischemic stroke. Neurological Res. (2016) 38:495–503. doi: 10.1080/01616412.2016.1188473, PMID: 27244271

[22] Lambertsen KL Biber K Finsen B. Inflammatory cytokines in experimental and human stroke. J Cereb Blood Flow metabolism: Off J Int Soc Cereb Blood Flow Metab. (2012) 32:1677–98. doi: 10.1038/jcbfm.2012.88, PMID: 22739623 PMC3434626

[23] Piepke M Clausen BH Ludewig P Vienhues JH Bedke T Javidi E. Interleukin-10 improves stroke outcome by controlling the detrimental Interleukin-17A response. J neuroinflammation. (2021) 18:265. doi: 10.1186/s12974-021-02316-7, PMID: 34772416 PMC8590298

[24] Arunachalam P Ludewig P Melich P Arumugam TV Gerloff C Prinz I. CCR6 (CC chemokine receptor 6) is essential for the migration of detrimental natural interleukin-17-producing γδ T cells in stroke. Stroke. (2017) 48:1957–65. doi: 10.1161/STROKEAHA.117.016753, PMID: 28611085

[25] Qu G Wang S Zhou Z Jiang D Liao A Luo J. Comparing mouse and human tissue-resident γδ T cells. Front Immunol. (2022) 13:891687. doi: 10.3389/fimmu.2022.891687, PMID: 35757696 PMC9215113

[26] McVay LD Carding SR. Extrathymic origin of human gamma delta T cells during fetal development. J Immunol (Baltimore Md: 1950). (1996) 157:2873–82. doi: 10.4049/jimmunol.157.7.2873, PMID: 8816392

[27] Paul S Singh AK Shilpi Lal G. Phenotypic and functional plasticity of gamma-delta (γδ) T cells in inflammation and tolerance. Int Rev Immunol. (2014) 33:537–58. doi: 10.3109/08830185.2013.863306, PMID: 24354324

[28] Heilig JS Tonegawa S. Diversity of murine gamma genes and expression in fetal and adult T lymphocytes. Nature. (1986) 322:836–40. doi: 10.1038/322836a0, PMID: 2943999

[29] Nielsen MM Witherden DA Havran WL. γδ T cells in homeostasis and host defence of epithelial barrier tissues. Nat Rev Immunol. (2017) 17:733–45. doi: 10.1038/nri.2017.101, PMID: 28920588 PMC5771804

[30] Roark CL French JD Taylor MA Bendele AM Born WK O'Brien RL. Exacerbation of collagen-induced arthritis by oligoclonal, IL-17-producing gamma delta T cells. J Immunol (Baltimore Md: 1950). (2007) 179:5576–83. doi: 10.4049/jimmunol.179.8.5576, PMID: 17911645 PMC2768546

[31] Ribot JC deBarros A Pang DJ Neves JF Peperzak V Roberts SJ. CD27 is a thymic determinant of the balance between interferon-gamma- and interleukin 17-producing gammadelta T cell subsets. Nat Immunol. (2009) 10:427–36. doi: 10.1038/ni.1717, PMID: 19270712 PMC4167721

[32] Kang J Volkmann A Raulet DH. Evidence that gammadelta versus alphabeta T cell fate determination is initiated independently of T cell receptor signaling. J Exp Med. (2001) 193:689–98. doi: 10.1084/jem.193.6.689, PMID: 11257136 PMC2193423

[33] Melichar HJ Narayan K Der SD Hiraoka Y Gardiol N Jeannet G. Regulation of gammadelta versus alphabeta T lymphocyte differentiation by the transcription factor SOX13. Sci (New York NY). (2007) 315:230–3. doi: 10.1126/science.1135344, PMID: 17218525

[34] Pizzolato G Kaminski H Tosolini M Franchini DM Pont F Martins F. Single-cell RNA sequencing unveils the shared and the distinct cytotoxic hallmarks of human TCRVδ1 and TCRVδ2 γδ T lymphocytes. Proc Natl Acad Sci United States America. (2019) 116:11906–15. doi: 10.1073/pnas.1818488116, PMID: 31118283 PMC6576116

[35] Song Z Henze L Casar C Schwinge D Schramm C Fuss J. Human γδ T cell identification from single-cell RNA sequencing datasets by modular TCR expression. J leukocyte Biol. (2023) 114:630–8. doi: 10.1093/jleuko/qiad069, PMID: 37437101

[36] Giri S Lal G. Differentiation and functional plasticity of gamma-delta (γδ) T cells under homeostatic and disease conditions. Mol Immunol. (2021) 136:138–49. doi: 10.1016/j.molimm.2021.06.006, PMID: 34146759

[37] Hayday AC. gamma][delta] cells: a right time and a right place for a conserved third way of protection. Annu Rev Immunol. (2000) 18:975–1026. doi: 10.1146/annurev.immunol.18.1.975, PMID: 10837080

[38] Swardfager W Winer DA Herrmann N Winer S Lanctôt KL. Interleukin-17 in post-stroke neurodegeneration. Neurosci Biobehav Rev. (2013) 37:436–47. doi: 10.1016/j.neubiorev.2013.01.021, PMID: 23370232

[39] Martin B Hirota K Cua DJ Stockinger B Veldhoen M. Interleukin-17-producing gammadelta T cells selectively expand in response to pathogen products and environmental signals. Immunity. (2009) 31:321–30. doi: 10.1016/j.immuni.2009.06.020, PMID: 19682928

[40] Piepke M Jander A Gagliani N Gelderblom M. IL-17A-producing gammadelta T cells: A novel target in stroke immunotherapy. Eur J Immunol. (2024) 54:e2451067. doi: 10.1002/eji.202451067, PMID: 39396374 PMC11628885

[41] Abou-El-Hassan H Rezende RM Izzy S Gabriely G Yahya T Tatematsu BK. Vgamma1 and Vgamma4 gamma-delta T cells play opposing roles in the immunopathology of traumatic brain injury in males. Nat Commun. (2023) 14:4286. doi: 10.1038/s41467-023-39857-9, PMID: 37463881 PMC10354011

[42] Li Y Zhu H Cheng D Zhao Z. Inhibition of gammadelta T cells alleviates brain ischemic injury in cardiopulmonary-cerebral resuscitation mice. Transplant Proc. (2022) 54:1984–91. doi: 10.1016/j.transproceed.2022.05.033, PMID: 35931471

[43] Frydrychowicz M Telec M Aniola J Kazmierski R Chowaniec H Dworacki G. The Alteration of Circulating Invariant Natural Killer T, gammadeltaT, and Natural Killer Cells after Ischemic Stroke in Relation to Clinical Outcomes: A Prospective Case-Control Study. Cells. (2024) 13:1401. doi: 10.3390/cells13161401, PMID: 39195289 PMC11352391

[44] Dobrota Lai S Buzkova P Delaney JA Olson NC Psaty BM Huber SA. Association of immune cell subsets with longevity: the cardiovascular health study. journals gerontology Ser A Biol Sci Med Sci. (2025) 80:glaf094. doi: 10.1093/gerona/glaf094, PMID: 40378276 PMC12159800

[45] Man SL Dong P Liu W Li HC Zhang L Ji XJ. Results of flow cytometric detection of gamma-deltaT cells in peripheral blood of patients with ankylosing spondylitis: a pilot study. Physiol Res. (2023) 72:819–32. doi: 10.33549/physiolres, PMID: 38215067 PMC10805258

[46] Hughes CE Nibbs RJB. A guide to chemokines and their receptors. FEBS J. (2018) 285:2944–71. doi: 10.1111/febs.2018.285.issue-16, PMID: 29637711 PMC6120486

[47] Monneau Y Arenzana-Seisdedos F Lortat-Jacob H. The sweet spot: how GAGs help chemokines guide migrating cells. J leukocyte Biol. (2016) 99:935–53. doi: 10.1189/jlb.3MR0915-440R, PMID: 26701132

[48] Salanga CL Handel TM. Chemokine oligomerization and interactions with receptors and glycosaminoglycans: the role of structural dynamics in function. Exp Cell Res. (2011) 317:590–601. doi: 10.1016/j.yexcr.2011.01.004, PMID: 21223963 PMC3089961

[49] Guo YQ Zheng LN Wei JF Hou XL Yu SZ Zhang WW. Expression of CCL2 and CCR2 in the hippocampus and the interventional roles of propofol in rat cerebral ischemia/reperfusion. Exp Ther Med. (2014) 8:657–61. doi: 10.3892/etm.2014.1757, PMID: 25009636 PMC4079442

[50] Joy MT Ben Assayag E Shabashov-Stone D Liraz-Zaltsman S Mazzitelli J Arenas M. CCR5 is a therapeutic target for recovery after stroke and traumatic brain injury. Cell. (2019) 176:1143–57.e13. doi: 10.1016/j.cell.2019.01.044, PMID: 30794775 PMC7259116

[51] Stumm RK Rummel J Junker V Culmsee C Pfeiffer M Krieglstein J. A dual role for the SDF-1/CXCR4 chemokine receptor system in adult brain: isoform-selective regulation of SDF-1 expression modulates CXCR4-dependent neuronal plasticity and cerebral leukocyte recruitment after focal ischemia. J neuroscience: Off J Soc Neurosci. (2002) 22:5865–78. doi: 10.1523/JNEUROSCI.22-14-05865.2002, PMID: 12122049 PMC6757949

[52] Benakis C Brea D Caballero S Faraco G Moore J Murphy M. Commensal microbiota affects ischemic stroke outcome by regulating intestinal γδ T cells. Nat Med. (2016) 22:516–23. doi: 10.1038/nm.4068, PMID: 27019327 PMC4860105

[53] Arya AK Hu B. Brain-gut axis after stroke. Brain circulation. (2018) 4:165–73. doi: 10.4103/bc.bc_32_18, PMID: 30693343 PMC6329216

[54] Li Y Li G Zhang J Wu X Chen X. The dual roles of human γδ T cells: anti-tumor or tumor-promoting. Front Immunol. (2020) 11:619954. doi: 10.3389/fimmu.2020.619954, PMID: 33664732 PMC7921733

[55] Cai Y Shen X Ding C Qi C Li K Li X. Pivotal role of dermal IL-17-producing γδ T cells in skin inflammation. Immunity. (2011) 35:596–610. doi: 10.1016/j.immuni.2011.08.001, PMID: 21982596 PMC3205267

[56] Gray EE Suzuki K Cyster JG. Cutting edge: Identification of a motile IL-17-producing gammadelta T cell population in the dermis. J Immunol (Baltimore Md: 1950). (2011) 186:6091–5. doi: 10.4049/jimmunol.1100427, PMID: 21536803 PMC3098921

[57] Sumaria N Roediger B Ng LG Qin J Pinto R Cavanagh LL. Cutaneous immunosurveillance by self-renewing dermal gammadelta T cells. J Exp Med. (2011) 208:505–18. doi: 10.1084/jem.20101824, PMID: 21339323 PMC3058585

[58] McKenzie DR Kara EE Bastow CR Tyllis TS Fenix KA Gregor CE. IL-17-producing γδ T cells switch migratory patterns between resting and activated states. Nat Commun. (2017) 8:15632. doi: 10.1038/ncomms15632, PMID: 28580944 PMC5465362

[59] Laidlaw BJ Gray EE Zhang Y Ramírez-Valle F Cyster JG. Sphingosine-1-phosphate receptor 2 restrains egress of γδ T cells from the skin. J Exp Med. (2019) 216:1487–96. doi: 10.1084/jem.20190114, PMID: 31160320 PMC6605748

[60] Ribot JC Lopes N Silva-Santos B. γδ T cells in tissue physiology and surveillance. Nat Rev Immunol. (2021) 21:221–32. doi: 10.1038/s41577-020-00452-4, PMID: 33057185

[61] Li GQ Xia J Zeng W Luo W Liu L Zeng X. The intestinal gammadelta T cells: functions in the gut and in the distant organs. Front Immunol. (2023) 14:1206299. doi: 10.3389/fimmu.2023.1206299, PMID: 37398661 PMC10311558

[62] Xie B Zhang Y Han M Wang M Yu Y Chen X. Reversal of the detrimental effects of social isolation on ischemic cerebral injury and stroke-associated pneumonia by inhibiting small intestinal gammadelta T-cell migration into the brain and lung. J Cereb Blood Flow metabolism: Off J Int Soc Cereb Blood Flow Metab. (2023) 43:1267–84. doi: 10.1177/0271678X231167946, PMID: 37017434 PMC10369145

[63] Gelderblom M Arunachalam P Magnus T. γδ T cells as early sensors of tissue damage and mediators of secondary neurodegeneration. Front Cell Neurosci. (2014) 8:368. doi: 10.3389/fncel.2014.00368, PMID: 25414640 PMC4220696

[64] Sutton CE Lalor SJ Sweeney CM Brereton CF Lavelle EC Mills KH. Interleukin-1 and IL-23 induce innate IL-17 production from gammadelta T cells, amplifying Th17 responses and autoimmunity. Immunity. (2009) 31:331–41. doi: 10.1016/j.immuni.2009.08.001, PMID: 19682929

[65] Han M Ma B She R Xing Y Li X. Correlations between serum CXCL9/12 and the severity of acute ischemic stroke, a retrospective observational study. Neuropsychiatr Dis Treat. (2023) 19:283–92. doi: 10.2147/NDT.S391578, PMID: 36744204 PMC9893834

[66] Liu L Yang C Lavayen BP Tishko RJ Larochelle J Candelario-Jalil E. Targeted BRD4 protein degradation by dBET1 ameliorates acute ischemic brain injury and improves functional outcomes associated with reduced neuroinflammation and oxidative stress and preservation of blood-brain barrier integrity. J neuroinflammation. (2022) 19:168. doi: 10.1186/s12974-022-02533-8, PMID: 35761277 PMC9237998

[67] Klimiec-Moskal E Koceniak P Weglarczyk K Slowik A Siedlar M Dziedzic T. Circulating chemokines and short- and long-term outcomes after ischemic stroke. Mol neurobiology. (2025) 62:421–8. doi: 10.1007/s12035-024-04279-1, PMID: 38861234 PMC11711783

[68] Wang L Yao C Chen J Ge Y Wang C Wang Y. γδ T cell in cerebral ischemic stroke: characteristic, immunity-inflammatory role, and therapy. Front neurology. (2022) 13:842212. doi: 10.3389/fneur.2022.842212, PMID: 35432162 PMC9008352

[69] Wang SW Liu Z Shi ZS. Non-coding RNA in acute ischemic stroke: mechanisms, biomarkers and therapeutic targets. Cell transplantation. (2018) 27:1763–77. doi: 10.1177/0963689718806818, PMID: 30362372 PMC6300774

[70] Jayaraj RL Azimullah S Beiram R Jalal FY Rosenberg GA. Neuroinflammation: friend and foe for ischemic stroke. J neuroinflammation. (2019) 16:142. doi: 10.1186/s12974-019-1516-2, PMID: 31291966 PMC6617684

[71] Wlodarczyk A Cédile O Jensen KN Jasson A Mony JT Khorooshi R. Pathologic and protective roles for microglial subsets and bone marrow- and blood-derived myeloid cells in central nervous system inflammation. Front Immunol. (2015) 6:463. doi: 10.3389/fimmu.2015.00463, PMID: 26441968 PMC4562247

[72] Colonna M Butovsky O. Microglia function in the central nervous system during health and neurodegeneration. Annu Rev Immunol. (2017) 35:441–68. doi: 10.1146/annurev-immunol-051116-052358, PMID: 28226226 PMC8167938

[73] Nimmerjahn A Kirchhoff F Helmchen F. Resting microglial cells are highly dynamic surveillants of brain parenchyma *in vivo* . Sci (New York NY). (2005) 308:1314–8. doi: 10.1126/science.1110647, PMID: 15831717

[74] Sierra A Encinas JM Deudero JJ Chancey JH Enikolopov G Overstreet-Wadiche LS. Microglia shape adult hippocampal neurogenesis through apoptosis-coupled phagocytosis. Cell Stem Cell. (2010) 7:483–95. doi: 10.1016/j.stem.2010.08.014, PMID: 20887954 PMC4008496

[75] Parkhurst CN Yang G Ninan I Savas JN Yates JR 3rd Lafaille JJ. Microglia promote learning-dependent synapse formation through brain-derived neurotrophic factor. Cell. (2013) 155:1596–609. doi: 10.1016/j.cell.2013.11.030, PMID: 24360280 PMC4033691

[76] Schafer DP Stevens B. Phagocytic glial cells: sculpting synaptic circuits in the developing nervous system. Curr Opin neurobiology. (2013) 23:1034–40. doi: 10.1016/j.conb.2013.09.012, PMID: 24157239 PMC3907950

[77] Davalos D Grutzendler J Yang G Kim JV Zuo Y Jung S. ATP mediates rapid microglial response to local brain injury *in vivo* . Nat Neurosci. (2005) 8:752–8. doi: 10.1038/nn1472, PMID: 15895084

[78] Ceulemans AG Zgavc T Kooijman R Hachimi-Idrissi S Sarre S Michotte Y. The dual role of the neuroinflammatory response after ischemic stroke: modulatory effects of hypothermia. J neuroinflammation. (2010) 7:74. doi: 10.1186/1742-2094-7-74, PMID: 21040547 PMC2988764

[79] Dirnagl U Klehmet J Braun JS Harms H Meisel C Ziemssen T. Stroke-induced immunodepression: experimental evidence and clinical relevance. Stroke. (2007) 38:770–3. doi: 10.1161/01.STR.0000251441.89665.bc, PMID: 17261736

[80] Wolf SA Boddeke HW Kettenmann H. Microglia in physiology and disease. Annu Rev Physiol. (2017) 79:619–43. doi: 10.1146/annurev-physiol-022516-034406, PMID: 27959620

[81] Dordoe C Wang X Lin P Wang Z Hu J Wang D. Non-mitogenic fibroblast growth factor 1 protects against ischemic stroke by regulating microglia/macrophage polarization through Nrf2 and NF-κB pathways. Neuropharmacology. (2022) 212:109064. doi: 10.1016/j.neuropharm.2022.109064, PMID: 35452626

[82] Scheid S Lejarre A Wollborn J Buerkle H Goebel U Ulbrich F. Argon preconditioning protects neuronal cells with a Toll-like receptor-mediated effect. Neural regeneration Res. (2023) 18:1371–7. doi: 10.4103/1673-5374.355978, PMID: 36453425 PMC9838174

[83] Xue K Qi M She T Jiang Z Zhang Y Wang X. Argon mitigates post-stroke neuroinflammation by regulating M1/M2 polarization and inhibiting NF-κB/NLRP3 inflammasome signaling. J Mol Cell Biol. (2023) 14:mjac077. doi: 10.1093/jmcb/mjac077, PMID: 36574951 PMC10165685

[84] Chen YH Wu KH Wu HP. Unraveling the complexities of toll-like receptors: from molecular mechanisms to clinical applications. Int J Mol Sci. (2024) 25:5037. doi: 10.3390/ijms25095037, PMID: 38732254 PMC11084218

[85] Silva CR Saraiva AL Rossato MF Trevisan G Oliveira SM. What do we know about Toll-Like Receptors Involvement in Gout Arthritis? Endocrine Metab Immune Disord Drug Targets. (2023) 23:446–57. doi: 10.2174/1871530322666220523145728, PMID: 35616672

[86] Kawai T Ikegawa M Ori D Akira S. Decoding Toll-like receptors: Recent insights and perspectives in innate immunity. Immunity. (2024) 57:649–73. doi: 10.1016/j.immuni.2024.03.004, PMID: 38599164

[87] Lehnardt S Massillon L Follett P Jensen FE Ratan R Rosenberg PA. Activation of innate immunity in the CNS triggers neurodegeneration through a Toll-like receptor 4-dependent pathway. Proc Natl Acad Sci United States America. (2003) 100:8514–9. doi: 10.1073/pnas.1432609100, PMID: 12824464 PMC166260

[88] Anttila JE Whitaker KW Wires ES Harvey BK Airavaara M. Role of microglia in ischemic focal stroke and recovery: focus on Toll-like receptors. Prog Neuropsychopharmacol Biol Psychiatry. (2017) 79:3–14. doi: 10.1016/j.pnpbp.2016.07.003, PMID: 27389423 PMC5214845

[89] Zhang CJ Jiang M Zhou H Liu W Wang C Kang Z. TLR-stimulated IRAKM activates caspase-8 inflammasome in microglia and promotes neuroinflammation. J Clin Invest. (2018) 128:5399–412. doi: 10.1172/JCI121901, PMID: 30372424 PMC6264724

[90] Wang H Yuan J Wang Y Chen J. To study the mechanism of panax notoginseng in the treatment of aspirin resistance in the secondary prevention of stroke based on TLR4/MyD88/NF-κB signaling pathway: A study protocol. Medicine. (2022) 101:e31919. doi: 10.1097/MD.0000000000031919, PMID: 36550905 PMC9771212

[91] Liu L Xu TC Zhao ZA Zhang NN Li J Chen HS. Toll-like receptor 4 signaling in neurons mediates cerebral ischemia/reperfusion injury. Mol neurobiology. (2023) 60:864–74. doi: 10.1007/s12035-022-03122-9, PMID: 36385232

[92] Lehnardt S Lehmann S Kaul D Tschimmel K Hoffmann O Cho S. Toll-like receptor 2 mediates CNS injury in focal cerebral ischemia. J neuroimmunology. (2007) 190:28–33. doi: 10.1016/j.jneuroim.2007.07.023, PMID: 17854911

[93] Jiang CT Wu WF Deng YH Ge JW. Modulators of microglia activation and polarization in ischemic stroke (Review). Mol Med Rep. (2020) 21:2006–18. doi: 10.3892/mmr.2020.11003, PMID: 32323760 PMC7115206

[94] Yu F Wang Y Stetler AR Leak RK Hu X Chen J. Phagocytic microglia and macrophages in brain injury and repair. CNS Neurosci Ther. (2022) 28:1279–93. doi: 10.1111/cns.13899, PMID: 35751629 PMC9344092

[95] Greter M Lelios I Croxford AL. Microglia versus myeloid cell nomenclature during brain inflammation. Front Immunol. (2015) 6:249. doi: 10.3389/fimmu.2015.00249, PMID: 26074918 PMC4443742

[96] Zhang G Li Q Tao W Qin P Chen J Yang H. Sigma-1 receptor-regulated efferocytosis by infiltrating circulating macrophages/microglial cells protects against neuronal impairments and promotes functional recovery in cerebral ischemic stroke. Theranostics. (2023) 13:543–59. doi: 10.7150/thno.77088, PMID: 36632219 PMC9830433

[97] Chen S Chen H Du Q Shen J. Targeting myeloperoxidase (MPO) mediated oxidative stress and inflammation for reducing brain ischemia injury: potential application of natural compounds. Front Physiol. (2020) 11:433. doi: 10.3389/fphys.2020.00433, PMID: 32508671 PMC7248223

[98] Peng L Hu G Yao Q Wu J He Z Law BY. Microglia autophagy in ischemic stroke: A double-edged sword. Front Immunol. (2022) 13:1013311. doi: 10.3389/fimmu.2022.1013311, PMID: 36466850 PMC9708732

[99] Lu X Zhang J Ding Y Wu J Chen G. Novel therapeutic strategies for ischemic stroke: recent insights into autophagy. Oxid Med Cell longevity. (2022) 2022:3450207. doi: 10.1155/2022/3450207, PMID: 35720192 PMC9200548

[100] Hu K Gao Y Chu S Chen N. Review of the effects and Mechanisms of microglial autophagy in ischemic stroke. Int immunopharmacology. (2022) 108:108761. doi: 10.1016/j.intimp.2022.108761, PMID: 35729827

[101] Huang F Luo L Wu Y Xia D Xu F Gao J. Trilobatin promotes angiogenesis after cerebral ischemia-reperfusion injury via SIRT7/VEGFA signaling pathway in rats. Phytotherapy research: PTR. (2022) 36:2940–51. doi: 10.1002/ptr.v36.7, PMID: 35537702

[102] Cheng CY Ho TY Hsiang CY Tang NY Hsieh CL Kao ST. Angelica sinensis Exerts Angiogenic and Anti-apoptotic Effects Against Cerebral Ischemia-Reperfusion Injury by Activating p38MAPK/HIF-1[Formula: see text]/VEGF-A Signaling in Rats. Am J Chin Med. (2017) 45:1683–708. doi: 10.1142/S0192415X17500914, PMID: 29121798

[103] Cheng CY Huang HC Kao ST Lee YC. Angelica sinensis extract promotes neuronal survival by enhancing p38 MAPK-mediated hippocampal neurogenesis and dendritic growth in the chronic phase of transient global cerebral ischemia in rats. J ethnopharmacology. (2021) 278:114301. doi: 10.1016/j.jep.2021.114301, PMID: 34090910

[104] Jiao Y Ren S Wang L Wu G. PPARγ/RAD21 alleviates peripheral secondary brain injury in rat cerebral hemorrhage model through promoting M2 polarization of microglial cells. Int immunopharmacology. (2023) 114:109572. doi: 10.1016/j.intimp.2022.109572, PMID: 36538854

[105] Armeli F Mengoni B Maggi E Mazzoni C Preziosi A Mancini P. Milmed yeast alters the LPS-induced M1 microglia cells to form M2 anti-inflammatory phenotype. Biomedicines. (2022) 10:3116. doi: 10.3390/biomedicines10123116, PMID: 36551872 PMC9776009

[106] Perego C Fumagalli S Zanier ER Carlino E Panini N Erba E. Macrophages are essential for maintaining a M2 protective response early after ischemic brain injury. Neurobiol disease. (2016) 96:284–93. doi: 10.1016/j.nbd.2016.09.017, PMID: 27697537

[107] Nagy AM Fekete R Horvath G Koncsos G Kriston C Sebestyen A. Versatility of microglial bioenergetic machinery under starving conditions. Biochim Biophys Acta Bioenergetics. (2018) 1859:201–14. doi: 10.1016/j.bbabio.2017.12.002, PMID: 29273412

[108] Kempermann G Gage FH Aigner L Song H Curtis MA Thuret S. Human adult neurogenesis: evidence and remaining questions. Cell Stem Cell. (2018) 23:25–30. doi: 10.1016/j.stem.2018.04.004, PMID: 29681514 PMC6035081

[109] Shichita T Sugiyama Y Ooboshi H Sugimori H Nakagawa R Takada I. Pivotal role of cerebral interleukin-17-producing gammadeltaT cells in the delayed phase of ischemic brain injury. Nat Med. (2009) 15:946–50. doi: 10.1038/nm.1999, PMID: 19648929

[110] Derkow K Krüger C Dembny P Lehnardt S. Microglia induce neurotoxic IL-17+ γδ T cells dependent on TLR2, TLR4, and TLR9 activation. PloS One. (2015) 10:e0135898. doi: 10.1371/journal.pone.0135898, PMID: 26288016 PMC4545749

[111] Zhang W Song J Li W Kong D Liang Y Zhao X. Salvianolic acid D alleviates cerebral ischemia-reperfusion injury by suppressing the cytoplasmic translocation and release of HMGB1-triggered NF-κB activation to inhibit inflammatory response. Mediators inflammation. (2020) 2020:9049614. doi: 10.1155/2020/9049614, PMID: 32410871 PMC7204335

[112] Chu J Li X Qu G Wang Y Li Q Guo Y. Chlamydia psittaci pmpD-N exacerbated chicken macrophage function by triggering th2 polarization and the TLR2/myD88/NF-κB signaling pathway. Int J Mol Sci. (2020) 21:2003. doi: 10.3390/ijms21062003, PMID: 32183481 PMC7139469

[113] Wang G Jin S Liu J Li X Dai P Wang Y. A neuron-immune circuit regulates neurodegeneration in the hindbrain and spinal cord of Arf1-ablated mice. Natl Sci Rev. (2023) 10:nwad222. doi: 10.1093/nsr/nwad222, PMID: 38239560 PMC10794899

[114] Xie L Sun F Wang J Mao X Xie L Yang SH. mTOR signaling inhibition modulates macrophage/microglia-mediated neuroinflammation and secondary injury via regulatory T cells after focal ischemia. J Immunol (Baltimore Md: 1950). (2014) 192:6009–19. doi: 10.4049/jimmunol.1303492, PMID: 24829408 PMC4128178

[115] Kuo PC Scofield BA Yu IC Chang FL Ganea D Yen JH. Interferon-β Modulates inflammatory response in cerebral ischemia. J Am Heart Assoc. (2016) 5:e002610. doi: 10.1161/JAHA.115.002610, PMID: 26747000 PMC4859377

[116] Pan Y Tian D Wang H Zhao Y Zhang C Wang S. Inhibition of perforin-mediated neurotoxicity attenuates neurological deficits after ischemic stroke. Front Cell Neurosci. (2021) 15:664312. doi: 10.3389/fncel.2021.664312, PMID: 34262436 PMC8274971

[117] Kauppinen TM Suh SW Berman AE Hamby AM Swanson RA. Inhibition of poly(ADP-ribose) polymerase suppresses inflammation and promotes recovery after ischemic injury. J Cereb Blood Flow metabolism: Off J Int Soc Cereb Blood Flow Metab. (2009) 29:820–9. doi: 10.1038/jcbfm.2009.9, PMID: 19190653

[118] Hayakawa K Mishima K Nozako M Hazekawa M Mishima S Fujioka M. Delayed treatment with minocycline ameliorates neurologic impairment through activated microglia expressing a high-mobility group box1-inhibiting mechanism. Stroke. (2008) 39:951–8. doi: 10.1161/STROKEAHA.107.495820, PMID: 18258837

[119] Kim HJ Rowe M Ren M Hong JS Chen PS Chuang DM. Histone deacetylase inhibitors exhibit anti-inflammatory and neuroprotective effects in a rat permanent ischemic model of stroke: multiple mechanisms of action. J Pharmacol Exp Ther. (2007) 321:892–901. doi: 10.1124/jpet.107.120188, PMID: 17371805

[120] Hayes SM Love PE. A retrospective on the requirements for gammadelta T-cell development. Immunol Rev. (2007) 215:8–14. doi: 10.1111/j.1600-065X.2006.00476.x, PMID: 17291275

[121] Fischer MA Golovchenko NB Edelblum KL. γδ T cell migration: Separating trafficking from surveillance behaviors at barrier surfaces. Immunol Rev. (2020) 298:165–80. doi: 10.1111/imr.v298.1, PMID: 32845516 PMC7968450

[122] Shibata K. Close link between development and function of gamma-delta T cells. Microbiol Immunol. (2012) 56:217–27. doi: 10.1111/j.1348-0421.2012.00435.x, PMID: 22300310

[123] McGraw JM Thelen F Hampton EN Bruno NE Young TS Havran WL. JAML promotes CD8 and γδ T cell antitumor immunity and is a novel target for cancer immunotherapy. J Exp Med. (2021) 218:e20202644. doi: 10.1084/jem.20202644, PMID: 34427588 PMC8404475

[124] Zarobkiewicz MK Morawska I Kowalska W Halczuk P Roliński J Bojarska-Junak AA. PECAM-1 Is Down-Regulated in γδT Cells during Remission, but Up-Regulated in Relapse of Multiple Sclerosis. J Clin Med. (2022) 11:3210. doi: 10.3390/jcm11113210, PMID: 35683597 PMC9181399

[125] Park JH Lee HK. Function of γδ T cells in tumor immunology and their application to cancer therapy. Exp Mol Med. (2021) 53:318–27. doi: 10.1038/s12276-021-00576-0, PMID: 33707742 PMC8080836

[126] McCarthy NE Eberl M. Human γδ T-cell control of mucosal immunity and inflammation. Front Immunol. (2018) 9:985. doi: 10.3389/fimmu.2018.00985, PMID: 29867962 PMC5949325

[127] Islam SA Chang DS Colvin RA Byrne MH McCully ML Moser B. Mouse CCL8, a CCR8 agonist, promotes atopic dermatitis by recruiting IL-5+ T(H)2 cells. Nat Immunol. (2011) 12:167–77. doi: 10.1038/ni.1984, PMID: 21217759 PMC3863381

[128] Poggi A Carosio R Fenoglio D Brenci S Murdaca G Setti M. Migration of V delta 1 and V delta 2 T cells in response to CXCR3 and CXCR4 ligands in healthy donors and HIV-1-infected patients: competition by HIV-1 Tat. Blood. (2004) 103:2205–13. doi: 10.1182/blood-2003-08-2928, PMID: 14630801

[129] Righi E Kashiwagi S Yuan J Santosuosso M Leblanc P Ingraham R. CXCL12/CXCR4 blockade induces multimodal antitumor effects that prolong survival in an immunocompetent mouse model of ovarian cancer. Cancer Res. (2011) 71:5522–34. doi: 10.1158/0008-5472.CAN-10-3143, PMID: 21742774 PMC3959864

[130] Liu Y Ran H Xiao Y Wang H Chen Y Chen W. Knockdown of HIF-1α impairs post-ischemic vascular reconstruction in the brain via deficient homing and sprouting bmEPCs. Brain Pathol (Zurich Switzerland). (2018) 28:860–74. doi: 10.1111/bpa.2018.28.issue-6, PMID: 30052311 PMC8028501

[131] ZAruba MM Staggl S Ghadge SK Maurer T Gavranovic-Novakovic J Jeyakumar V. Roxadustat attenuates adverse remodeling following myocardial infarction in mice. Cells. (2024) 13:1074. doi: 10.3390/cells13131074, PMID: 38994928 PMC11240812

[132] Ponomarev ED Novikova M Yassai M Szczepanik M Gorski J Dittel BN. Gamma delta T cell regulation of IFN-gamma production by central nervous system-infiltrating encephalitogenic T cells: correlation with recovery from experimental autoimmune encephalomyelitis. J Immunol (Baltimore Md: 1950). (2004) 173:1587–95. doi: 10.4049/jimmunol.173.3.1587, PMID: 15265886

[133] Maimaitijiang G Watanabe M Shinoda K Isobe N Nakamura Y Masaki K. Long-term use of interferon-β in multiple sclerosis increases Vδ1(-)Vδ2(-)Vγ9(-) γδ T cells that are associated with a better outcome. J neuroinflammation. (2019) 16:179. doi: 10.1186/s12974-019-1574-5, PMID: 31519178 PMC6743159

[134] McGinley AM Sutton CE Edwards SC Leane CM DeCourcey J Teijeiro A. Interleukin-17A serves a priming role in autoimmunity by recruiting IL-1β-producing myeloid cells that promote pathogenic T cells. Immunity. (2020) 52:342–56.e6. doi: 10.1016/j.immuni.2020.01.002, PMID: 32023490

[135] Wohler JE Smith SS Zinn KR Bullard DC Barnum SR. Gammadelta T cells in EAE: early trafficking events and cytokine requirements. Eur J Immunol. (2009) 39:1516–26. doi: 10.1002/eji.200839176, PMID: 19384874 PMC2837942

[136] Gelderblom M Weymar A Bernreuther C Velden J Arunachalam P Steinbach K. Neutralization of the IL-17 axis diminishes neutrophil invasion and protects from ischemic stroke. Blood. (2012) 120:3793–802. doi: 10.1182/blood-2012-02-412726, PMID: 22976954

[137] Lin Y Zhang JC Yao CY Wu Y Abdelgawad AF Yao SL. Critical role of astrocytic interleukin-17 A in post-stroke survival and neuronal differentiation of neural precursor cells in adult mice. Cell Death disease. (2016) 7:e2273. doi: 10.1038/cddis.2015.284, PMID: 27336717 PMC5143370

[138] Burke SJ Lu D Sparer TE Masi T Goff MR Karlstad MD. NF-κB and STAT1 control CXCL1 and CXCL2 gene transcription. Am J Physiol Endocrinol Metab. (2014) 306:E131–49. doi: 10.1152/ajpendo.00347.2013, PMID: 24280128 PMC3920007

[139] Honda K Wada H Nakamura M Nakamoto K Inui T Sada M. IL-17A synergistically stimulates TNF-α-induced IL-8 production in human airway epithelial cells: A potential role in amplifying airway inflammation. Exp Lung Res. (2016) 42:205–16. doi: 10.1080/01902148.2016.1190796, PMID: 27269887

[140] Pillay J Kamp VM van Hoffen E Visser T Tak T Lammers JW. A subset of neutrophils in human systemic inflammation inhibits T cell responses through Mac-1. J Clin Invest. (2012) 122:327–36. doi: 10.1172/JCI57990, PMID: 22156198 PMC3248287

[141] O'Brien RL Yin X Huber SA Ikuta K Born WK. Depletion of a gamma delta T cell subset can increase host resistance to a bacterial infection. J Immunol (Baltimore Md: 1950). (2000) 165:6472–9. doi: 10.4049/jimmunol.165.11.6472, PMID: 11086087

[142] Gelderblom M Gallizioli M Ludewig P Thom V Arunachalam P Rissiek B. IL-23 (Interleukin-23)-producing conventional dendritic cells control the detrimental IL-17 (Interleukin-17) response in stroke. Stroke. (2018) 49:155–64. doi: 10.1161/STROKEAHA.117.019101, PMID: 29212740

[143] Ruhnau J Schulze J Dressel A Vogelgesang A. Thrombosis, neuroinflammation, and poststroke infection: the multifaceted role of neutrophils in stroke. J Immunol Res. (2017) 2017:5140679. doi: 10.1155/2017/5140679, PMID: 28331857 PMC5346374

[144] Kumari R Sinha K. Neutrophil in diabetic stroke: emerging therapeutic strategies. Neural regeneration Res. (2021) 16:2206–8. doi: 10.4103/1673-5374.310677, PMID: 33818495 PMC8354114

[145] Shi Y Zhang L Pu H Mao L Hu X Jiang X. Rapid endothelial cytoskeletal reorganization enables early blood-brain barrier disruption and long-term ischaemic reperfusion brain injury. Nat Commun. (2016) 7:10523. doi: 10.1038/ncomms10523, PMID: 26813496 PMC4737895

[146] Zhang S An Q Wang T Gao S Zhou G. Autophagy- and MMP-2/9-mediated reduction and redistribution of ZO-1 contribute to hyperglycemia-increased blood-brain barrier permeability during early reperfusion in stroke. Neuroscience. (2018) 377:126–37. doi: 10.1016/j.neuroscience.2018.02.035, PMID: 29524637

[147] Yang Y Thompson JF Taheri S Salayandia VM McAvoy TA Hill JW. Early inhibition of MMP activity in ischemic rat brain promotes expression of tight junction proteins and angiogenesis during recovery. J Cereb Blood Flow metabolism: Off J Int Soc Cereb Blood Flow Metab. (2013) 33:1104–14. doi: 10.1038/jcbfm.2013.56, PMID: 23571276 PMC3705440

[148] Dhanesha N Patel RB Doddapattar P Ghatge M Flora GD Jain M. PKM2 promotes neutrophil activation and cerebral thromboinflammation: therapeutic implications for ischemic stroke. Blood. (2022) 139:1234–45. doi: 10.1182/blood.2021012322, PMID: 34529778 PMC8874361

[149] Carbone F Bonaventura A Montecucco F. Neutrophil-related oxidants drive heart and brain remodeling after ischemia/reperfusion injury. Front Physiol. (2019) 10:1587. doi: 10.3389/fphys.2019.01587, PMID: 32116732 PMC7010855

[150] Denorme F Portier I Rustad JL Cody MJ de Araujo CV Hoki C. Neutrophil extracellular traps regulate ischemic stroke brain injury. J Clin Invest. (2022) 132:e154225. doi: 10.1172/JCI154225, PMID: 35358095 PMC9106355

[151] Arnholdt C Kumaraswami K Götz P Kübler M Lasch M Deindl E. Depletion of γδ T cells leads to reduced angiogenesis and increased infiltration of inflammatory M1-like macrophages in ischemic muscle tissue. Cells. (2022) 11:1490. doi: 10.3390/cells11091490, PMID: 35563796 PMC9102774

[152] Kang L Yu H Yang X Zhu Y Bai X Wang R. Neutrophil extracellular traps released by neutrophils impair revascularization and vascular remodeling after stroke. Nat Commun. (2020) 11:2488. doi: 10.1038/s41467-020-16191-y, PMID: 32427863 PMC7237502

[153] Xie M Hao Y Feng L Wang T Yao M Li H. Neutrophil Heterogeneity and its Roles in the Inflammatory Network after Ischemic Stroke. Curr neuropharmacology. (2023) 21:621–50. doi: 10.2174/1570159X20666220706115957, PMID: 35794770 PMC10207908

[154] Pektezel MY Yilmaz E Arsava EM Topcuoglu MA. Neutrophil-to-lymphocyte ratio and response to intravenous thrombolysis in patients with acute ischemic stroke. J stroke cerebrovascular diseases: Off J Natl Stroke Assoc. (2019) 28:1853–9. doi: 10.1016/j.jstrokecerebrovasdis.2019.04.014, PMID: 31072698

[155] Blank-Stein N Mass E. Macrophage and monocyte subsets in response to ischemic stroke. Eur J Immunol. (2023) 53:e2250233. doi: 10.1002/eji.202250233, PMID: 37467166

[156] Miró-Mur F Pérez-de-Puig I Ferrer-Ferrer M Urra X Justicia C Chamorro A. Immature monocytes recruited to the ischemic mouse brain differentiate into macrophages with features of alternative activation. Brain behavior immunity. (2016) 53:18–33. doi: 10.1016/j.bbi.2015.08.010, PMID: 26275369

[157] Werner Y Mass E Ashok Kumar P Ulas T Händler K Horne A. Cxcr4 distinguishes HSC-derived monocytes from microglia and reveals monocyte immune responses to experimental stroke. Nat Neurosci. (2020) 23:351–62. doi: 10.1038/s41593-020-0585-y, PMID: 32042176 PMC7523735

[158] Barin JG Baldeviano GC Talor MV Wu L Ong S Quader F. Macrophages participate in IL-17-mediated inflammation. Eur J Immunol. (2012) 42:726–36. doi: 10.1002/eji.201141737, PMID: 22161142 PMC4292791

[159] Nordlohne J von Vietinghoff S. Interleukin 17A in atherosclerosis - Regulation and pathophysiologic effector function. Cytokine. (2019) 122:154089. doi: 10.1016/j.cyto.2017.06.016, PMID: 28663097

[160] Han D Liu H Gao Y. The role of peripheral monocytes and macrophages in ischemic stroke. Neurological sciences: Off J Ital Neurological Soc Ital Soc Clin Neurophysiology. (2020) 41:3589–607. doi: 10.1007/s10072-020-04777-9, PMID: 33009963

[161] Shekhar S Cunningham MW Pabbidi MR Wang S Booz GW Fan F. Targeting vascular inflammation in ischemic stroke: Recent developments on novel immunomodulatory approaches. Eur J Pharmacol. (2018) 833:531–44. doi: 10.1016/j.ejphar.2018.06.028, PMID: 29935175 PMC6090562

[162] Wan J Zhang Q Hao Y Tao Z Song W Chen S. Infiltrated IL-17A-producing gamma delta T cells play a protective role in sepsis-induced liver injury and are regulated by CCR6 and gut commensal microbes. Front Cell infection Microbiol. (2023) 13:1149506. doi: 10.3389/fcimb.2023.1149506, PMID: 37475963 PMC10354519

[163] Wu S Xie Y Jiang Y Zhang X Zhou Y Zuo X. GTS-21 modulates rheumatoid arthritis Th17 and Th2 lymphocyte subset differentiation through the α7nAch receptor. Clin Rheumatol. (2025) 44:989–98. doi: 10.1007/s10067-025-07320-3, PMID: 39812970

[164] Piepke M Jander A Gagliani N Gelderblom M. IL-17A-producing γδ T cells: A novel target in stroke immunotherapy. Eur J Immunol. (2024) 54:e2451067. doi: 10.1002/eji.202451067, PMID: 39396374 PMC11628885

[165] Wang J Gao Y Yuan Y Wang H Wang Z Zhang X. Th17 cells and IL-17A in ischemic stroke. Mol neurobiology. (2024) 61:2411–29. doi: 10.1007/s12035-023-03723-y, PMID: 37884768 PMC10973033

[166] Lian Z Luo Y Li Y Gao Y Xiong X Gu L. CD4(+) T cells in ischemic stroke: effects and therapeutic targets. Front Immunol. (2025) 16:1512634. doi: 10.3389/fimmu.2025.1512634, PMID: 40352928 PMC12061934

[167] Chen X Zhang Y Ding Q He Y Li H. Role of IL-17A in different stages of ischemic stroke. Int immunopharmacology. (2023) 117:109926. doi: 10.1016/j.intimp.2023.109926, PMID: 37012860

[168] Zheng Y Ren Z Liu Y Yan J Chen C He Y. T cell interactions with microglia in immune-inflammatory processes of ischemic stroke. Neural regeneration Res. (2025) 20:1277–92. doi: 10.4103/NRR.NRR-D-23-01385, PMID: 39075894 PMC11624874

[169] Navarro-Compán V Puig L Vidal S Ramírez J Llamas-Velasco M Fernández-Carballido C. The paradigm of IL-23-independent production of IL-17F and IL-17A and their role in chronic inflammatory diseases. Front Immunol. (2023) 14:1191782. doi: 10.3389/fimmu.2023.1191782, PMID: 37600764 PMC10437113

[170] Kinzel O Goldberg SD Cummings MD Gege C Steeneck C Xue X. Identification of JNJ-61803534, a RORγt inverse agonist for the treatment of psoriasis. J medicinal Chem. (2025) 68:8713–28. doi: 10.1021/acs.jmedchem.5c00390, PMID: 40237323

[171] Hamada S Umemura M Shiono T Tanaka K Yahagi A Begum MD. IL-17A produced by gammadelta T cells plays a critical role in innate immunity against listeria monocytogenes infection in the liver. J Immunol (Baltimore Md: 1950). (2008) 181:3456–63. doi: 10.4049/jimmunol.181.5.3456, PMID: 18714018 PMC2859669

[172] Viel S Marçais A Guimaraes FS Loftus R Rabilloud J Grau M. TGF-β inhibits the activation and functions of NK cells by repressing the mTOR pathway. Sci Signaling. (2016) 9:ra19. doi: 10.1126/scisignal.aad1884, PMID: 26884601

[173] Mirabelli-Badenier M Braunersreuther V Viviani GL Dallegri F Quercioli A Veneselli E. CC and CXC chemokines are pivotal mediators of cerebral injury in ischaemic stroke. Thromb haemostasis. (2011) 105:409–20. doi: 10.3390/cells11030491, PMID: 21174009

[174] Henderson SR Horsley H Frankel P Khosravi M Goble T Carter S. Proteinase 3 promotes formation of multinucleated giant cells and granuloma-like structures in patients with granulomatosis with polyangiitis. Ann rheumatic diseases. (2023) 82:848–56. doi: 10.1136/ard-2021-221800, PMID: 36801813 PMC10314067

[175] Muhammad S Chaudhry SR Kahlert UD Niemelä M Hänggi D. Brain immune interactions-novel emerging options to treat acute ischemic brain injury. Cells. (2021) 10:2429. doi: 10.3390/cells10092429, PMID: 34572077 PMC8472028

[176] Kim E Yang J Beltran CD Cho S. Role of spleen-derived monocytes/macrophages in acute ischemic brain injury. J Cereb Blood Flow metabolism: Off J Int Soc Cereb Blood Flow Metab. (2014) 34:1411–9. doi: 10.1038/jcbfm.2014.101, PMID: 24865998 PMC4126087

[177] Ran Y Su W Gao F Ding Z Yang S Ye L. Curcumin Ameliorates White Matter Injury after Ischemic Stroke by Inhibiting Microglia/Macrophage Pyroptosis through NF-κB Suppression and NLRP3 Inflammasome Inhibition. Oxid Med Cell longevity. (2021) 2021:1552127. doi: 10.1155/2021/1552127, PMID: 34630845 PMC8497115

[178] Zhu H Jian Z Zhong Y Ye Y Zhang Y Hu X. Janus kinase inhibition ameliorates ischemic stroke injury and neuroinflammation through reducing NLRP3 inflammasome activation via JAK2/STAT3 pathway inhibition. Front Immunol. (2021) 12:714943. doi: 10.3389/fimmu.2021.714943, PMID: 34367186 PMC8339584

[179] Kim DG Krenz A Toussaint LE Maurer KJ Robinson SA Yan A. Non-alcoholic fatty liver disease induces signs of Alzheimer's disease (AD) in wild-type mice and accelerates pathological signs of AD in an AD model. J neuroinflammation. (2016) 13:1. doi: 10.1186/s12974-015-0467-5, PMID: 26728181 PMC4700622

[180] Lei TY Ye YZ Zhu XQ Smerin D Gu LJ Xiong XX. The immune response of T cells and therapeutic targets related to regulating the levels of T helper cells after ischaemic stroke. J neuroinflammation. (2021) 18:25. doi: 10.1186/s12974-020-02057-z, PMID: 33461586 PMC7814595

[181] Liu F Cheng X Zhong S Liu C Jolkkonen J Zhang X. Communications between peripheral and the brain-resident immune system in neuronal regeneration after stroke. Front Immunol. (2020) 11:1931. doi: 10.3389/fimmu.2020.01931, PMID: 33042113 PMC7530165

[182] Liu Q Sorooshyari SK. Quantitative and correlational analysis of brain and spleen immune cellular responses following cerebral ischemia. Front Immunol. (2021) 12:617032. doi: 10.3389/fimmu.2021.617032, PMID: 34194419 PMC8238006

[183] Ngwa C Al Mamun A Qi S Sharmeen R Xu Y Liu F. Regulation of microglial activation in stroke in aged mice: a translational study. Aging. (2022) 14:6047–65. doi: 10.18632/aging.204216, PMID: 35963621 PMC9417226

[184] Su X Yang S Li Y Xiang Z Tao Q Liu S. γδ T cells recruitment and local proliferation in brain parenchyma benefit anti-neuroinflammation after cerebral microbleeds. Front Immunol. (2023) 14:1139601. doi: 10.3389/fimmu.2023.1139601, PMID: 37063908 PMC10090560

[185] Nguyen CT Maverakis E Eberl M Adamopoulos IE. γδ T cells in rheumatic diseases: from fundamental mechanisms to autoimmunity. Semin immunopathology. (2019) 41:595–605. doi: 10.1007/s00281-019-00752-5, PMID: 31506867 PMC6815259

[186] Edwards SC Sutton CE Ladell K Grant EJ McLaren JE Roche F. A population of proinflammatory T cells coexpresses αβ and γδ T cell receptors in mice and humans. J Exp Med. (2020) 217:e20190834. doi: 10.1084/jem.20190834, PMID: 32106283 PMC7201916

[187] Bhat J Placek K Faissner S. Contemplating dichotomous nature of gamma delta T cells for immunotherapy. Front Immunol. (2022) 13:894580. doi: 10.3389/fimmu.2022.894580, PMID: 35669772 PMC9163397

[188] Zhang Y Liesz A Li P. Coming to the rescue: regulatory T cells for promoting recovery after ischemic stroke. Stroke. (2021) 52:e837–e41. doi: 10.1161/STROKEAHA.121.036072, PMID: 34807742

[189] Wang HY Ye JR Cui LY Chu SF Chen NH. Regulatory T cells in ischemic stroke. Acta pharmacologica Sinica. (2022) 43:1–9. doi: 10.1038/s41401-021-00641-4, PMID: 33772140 PMC8724273

[190] Benakis C Simats A Tritschler S Heindl S Besson-Girard S Llovera G. T cells modulate the microglial response to brain ischemia. eLife. (2022) 11:e82031. doi: 10.7554/eLife.82031.sa2, PMID: 36512388 PMC9747154

[191] Cai W Shi L Zhao J Xu F Dufort C Ye Q. Neuroprotection against ischemic stroke requires a specific class of early responder T cells in mice. J Clin Invest. (2022) 132:e157678. doi: 10.1172/JCI157678, PMID: 35912857 PMC9337834

[192] Frydrychowicz M Telec M Anioła J Kazmierski R Chowaniec H Dworacki G. The alteration of circulating invariant natural killer T, γδT, and natural killer cells after ischemic stroke in relation to clinical outcomes: A prospective case-control study. Cells. (2024) 13:1401. doi: 10.3390/cells13161401, PMID: 39195289 PMC11352391

[193] Zhang J Mao X Zhou T Cheng X Lin Y. IL-17A contributes to brain ischemia reperfusion injury through calpain-TRPC6 pathway in mice. Neuroscience. (2014) 274:419–28. doi: 10.1016/j.neuroscience.2014.06.001, PMID: 24928352

[194] Godfrey DI Le Nours J Andrews DM Uldrich AP Rossjohn J. Unconventional T cell targets for cancer immunotherapy. Immunity. (2018) 48:453–73. doi: 10.1016/j.immuni.2018.03.009, PMID: 29562195

[195] Corbett AJ Awad W Wang H Chen Z. Antigen recognition by MR1-reactive T cells; MAIT cells, metabolites, and remaining mysteries. Front Immunol. (2020) 11:1961. doi: 10.3389/fimmu.2020.01961, PMID: 32973800 PMC7482426

[196] Silva-Santos B Mensurado S Coffelt SB. γδ T cells: pleiotropic immune effectors with therapeutic potential in cancer. Nat Rev Cancer. (2019) 19:392–404. doi: 10.1038/s41568-019-0153-5, PMID: 31209264 PMC7614706

[197] Xiao T Sun M Kang J Zhao C. Transient receptor potential vanilloid1 (TRPV1) channel opens sesame of T cell responses and T cell-mediated inflammatory diseases. Front Immunol. (2022) 13:870952. doi: 10.3389/fimmu.2022.870952, PMID: 35634308 PMC9130463

[198] Froghi S Grant CR Tandon R Quaglia A Davidson B Fuller B. New insights on the role of TRP channels in calcium signalling and immunomodulation: review of pathways and implications for clinical practice. Clin Rev Allergy Immunol. (2021) 60:271–92. doi: 10.1007/s12016-020-08824-3, PMID: 33405100 PMC7985118

[199] Huang Q Wang X Lin X Zhang J You X Shao A. The role of transient receptor potential channels in blood-brain barrier dysfunction after ischemic stroke. Biomedicine pharmacotherapy = Biomedecine pharmacotherapie. (2020) 131:110647. doi: 10.1016/j.biopha.2020.110647, PMID: 32858500

[200] Zong P Li CX Feng J Cicchetti M Yue L. TRP channels in stroke. Neurosci bulletin. (2024) 40:1141–59. doi: 10.1007/s12264-023-01151-5, PMID: 37995056 PMC11306852

[201] Chen X Zhang J Wang K. Inhibition of intracellular proton-sensitive Ca(2+)-permeable TRPV3 channels protects against ischemic brain injury. Acta Pharm Sin B. (2022) 12:2330–47. doi: 10.1016/j.apsb.2022.01.001, PMID: 35646518 PMC9136580

[202] Zong P Lin Q Feng J Yue L. A systemic review of the integral role of TRPM2 in ischemic stroke: from upstream risk factors to ultimate neuronal death. Cells. (2022) 11:491. doi: 10.3390/cells11030491, PMID: 35159300 PMC8834171

[203] Goldberg EL Molony RD Kudo E Sidorov S Kong Y Dixit VD. Ketogenic diet activates protective γδ T cell responses against influenza virus infection. Sci Immunol. (2019) 4:eaav2026. doi: 10.1126/sciimmunol.aav2026, PMID: 31732517 PMC7189564

[204] Siracusa F Schaltenberg N Kumar Y Lesker TR Steglich B Liwinski T. Short-term dietary changes can result in mucosal and systemic immune depression. Nat Immunol. (2023) 24:1473–86. doi: 10.1038/s41590-023-01587-x, PMID: 37580603 PMC10457203

[205] Sullivan ZA Khoury-Hanold W Lim J Smillie C Biton M Reis BS. γδ T cells regulate the intestinal response to nutrient sensing. Science. (2021) 371:eaba8310. doi: 10.1126/science.aba8310, PMID: 33737460 PMC11617329

[206] Wang Y Chen Y Meng L Wu B Ouyang L Peng R. Electro-acupuncture treatment inhibits the inflammatory response by regulating γδ T and Treg cells in ischemic stroke. Exp Neurol. (2023) 362:114324. doi: 10.1016/j.expneurol.2023.114324, PMID: 36669751

